# ^1^H-NMR-Based Analysis for Exploring Knee Synovial Fluid Metabolite Changes after Local Cryotherapy in Knee Arthritis Patients

**DOI:** 10.3390/metabo10110460

**Published:** 2020-11-13

**Authors:** Wafa Douzi, Xavier Guillot, Delphine Bon, François Seguin, Nadège Boildieu, Daniel Wendling, Nicolas Tordi, Olivier Dupuy, Benoit Dugué

**Affiliations:** 1 Laboratoire «Mobilité, Vieillissement, Exercice (MOVE)–EA6314», Faculté des Sciences du Sport, Université de Poitiers, 8 Allée Jean Monnet, 86000 Poitiers, France; wafa.douzi01@univ-poitiers.fr (W.D.); olivier.dupuy@univ-poitiers.fr (O.D.); 2 Department of Rheumatology, Felix Guyon University Hospital, 97400 Saint-Denis, France; xavier.guillot@chu-reunion.fr; 3 INSERM U1082, (IRTOMIT), Poitiers, France and Faculty of Medicine and Pharmacy, University of Poitiers, 86000 Poitiers, France; delphine.bon@univ-poitiers.fr (D.B.); francois.seguin@univ-poitiers.fr (F.S.); nadege.boildieu@univ-poitiers.fr (N.B.); 4 Department of Rheumatology, CHRU de Besançon, Boulevard Fleming, F-25030 Besançon, France; dwendling@chu-besancon.fr; 5 PEPITE EA4267, (EPSI), University Bourgogne Franche-Comté, F-25000 Besançon, France; nicolas.tordi@univ-fcomte.fr

**Keywords:** metabolites, nuclear magnetic resonance spectroscopy, cryotherapy, synovial inflammation, arthritis

## Abstract

Rehabilitation using cryotherapy has widely been used in inflammatory diseases to relieve pain and decrease the disease activity. The aim of this study was to explore the metabolite changes in inflammatory knee-joint synovial fluids following local cryotherapy treatment (ice or cold CO_2_). We used proton nuclear magnetic resonance (^1^H NMR) spectroscopy to assess the metabolite patterns in synovial fluid (SF) in patients with knee arthritis (*n* = 46) before (D0) and after (D1, 24 h later) two applications of local cryotherapy. Spectra from aqueous samples and organic extracts were obtained with an 11.75 Tesla spectrometer. The metabolite concentrations within the SF were compared between D1 and D0 using multiple comparisons with the application of a false discovery rate (FDR) adjusted at 10% for each metabolite. A total of 32 metabolites/chemical structures were identified including amino acids, organic acids, fatty acids or sugars. Pyruvate, alanine, citrate, threonine was significantly higher at D1 vs D0 (*p* < 0.05). Tyrosine concentration significantly decreases after cryotherapy application (*p* < 0.001). We did not observe any effect of gender and cooling technique on metabolite concentrations between D0 and D1 (*p* > 0.05). The present study provides new insight into a short-term effect of cold stimulus in synovial fluid from patients with knee arthritis. Our observations suggest that the increased level of metabolites involved in energy metabolism may explain the underlying molecular pathways that mediate the antioxidant and anti-inflammatory capacities of cryotherapy.

## 1. Introduction

Inflammatory arthritis is characterized by an inflammation of the synovial membrane (synovitis), which leads to disability and a substantial reduction in quality of life [1]. The inflamed synovium contains infiltrated inflammatory cells (activated T cells and macrophages), as well as an excess of pro-inflammatory cytokines (interleukin-1 (IL-1) and Tumor Necrosis Factor alpha (TNFα)), accelerating thereby the production of reactive oxygen species (ROS) and connective tissue damages [2]. The raised ROS levels can damage tissues, proteins, lipids and matrix components [3]. Further, several studies have shown a raised oxidative stress in synovial fluid in rheumatoid arthritis patients and associated the disease to a declined antioxidant capacity [4]. The impaired antioxidant system amplifies the synovial inflammatory-proliferative response [4], and may provoke a joint degradation and periarticular deformities [5]. Without treatment, synovitis may lead to the destruction of articular cartilage and subchondral bone.

The symptomatic medications for inflammatory rheumatic diseases, including corticosteroids and nonsteroidal anti-inflammatory drugs (NSAIDs), are closely related to the oxidant/antioxidant imbalance and the inflammatory responses [4]. Synthetic disease modifying anti rheumatic drugs or targeted biologics may suppress disease inflammatory activity in rheumatoid arthritis or spondyloarthritis. However, pharmacotherapy alone is not enough to restore complete locomotor functions in patients suffering from knee arthritis. To reduce the oxidant stress and relieve pain in inflammatory diseases, adjuvant non-pharmacological methods are often used, such as cryotherapy [6,7]. It consists of an external application of cold (cooled gas, cooled fluids, ice) in the proximity of the joints. In addition to the low cost, this therapy is well known as efficient method to increase antioxidative buffering capacities [8,9] and to mitigate the symptoms of inflammation, edema, pain and swelling [10]. Cold-induced vasoconstriction induces a decrease in synovial blood flow in arthritic patients [11], reducing thereby the nerve conduction velocity, the nociceptor excitability thresholds, the local inflammatory mediators [12,13] and the enzyme activities [14]. Therefore, several studies suggested the use of cryotherapy as adjuvant therapy in patients with inflammatory diseases to improve the locomotor function and to reduce the disease activity [15,16,17].

Guillot et al. have carried out a series of studies in patients with knee arthritis [6,18] and arthritic rats [19] to investigate the impact of local cryotherapy on inflammatory mediators and enzyme pathways in the synovial fluid. The authors demonstrated that local cryotherapy (ice and cold CO_2_), applied twice at an interval of 8 h, inhibits pro-inflammatory cytokine and enzyme pathways in the synovial fluid of non-septic arthritic knees through a reduction in synovial levels of interleukin 6 (IL-6), interleukin 1β (IL-1β), vascular endothelial growth factor (VEGF), prostaglandin-E2 (PG-E2), and nuclear factor kappa B p65 (NF-kB-p65) [6]. They also reported that local cryotherapy reduces the synovial Power Doppler activity and pain over 24 h in knee arthritis [18]. Likewise, for the animal model, the authors have observed that local cryotherapy is efficient to reduce the disease activity through a down-regulation of joint and systemic IL-6/IL-17 pathway [19]. The current study comes as part of a continuity of these studies in the same group of patients suffering from knee arthritis.

We aimed to investigate the impact of local cryotherapy (ice and cold CO_2_), applied twice during 1 day at an 8-h interval, on metabolite content of the synovial fluid. To this end, we have used proton nuclear magnetic resonance (^1^H NMR) spectroscopy to identify the metabolite patterns in synovial fluid in patients with knee arthritis. Nuclear magnetic resonance (NMR) is a reproducible tool in analytical chemistry. It is used to assess and quantify a wide variety of metabolites in order to correctly diagnose diseases and reveal new therapeutic avenues [20,21,22]. This approach has been previously used to assess the metabolite profiles of knee-joint synovial fluid obtained from patients with knee arthritis [23,24,25] or from animal models of osteoarthritis [26,27]. The present study may provide novel insights into the underlying molecular pathways involved in cryotherapy’s antioxidative and anti-inflammatory effects.

## 2. Results

### 2.1. Metabolite Identification in SF Samples

**Figure 1:**
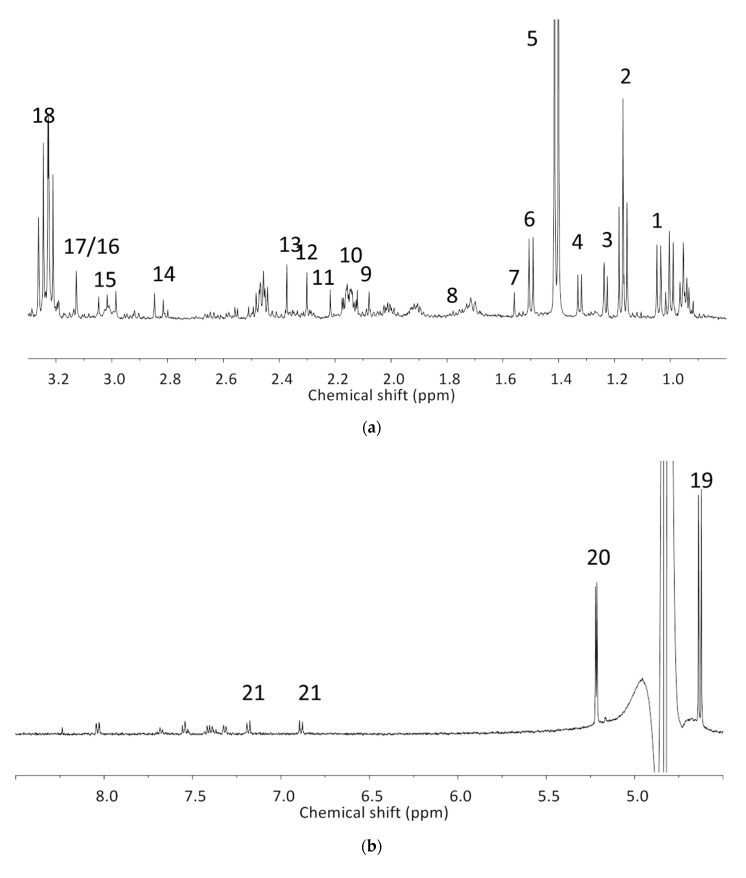

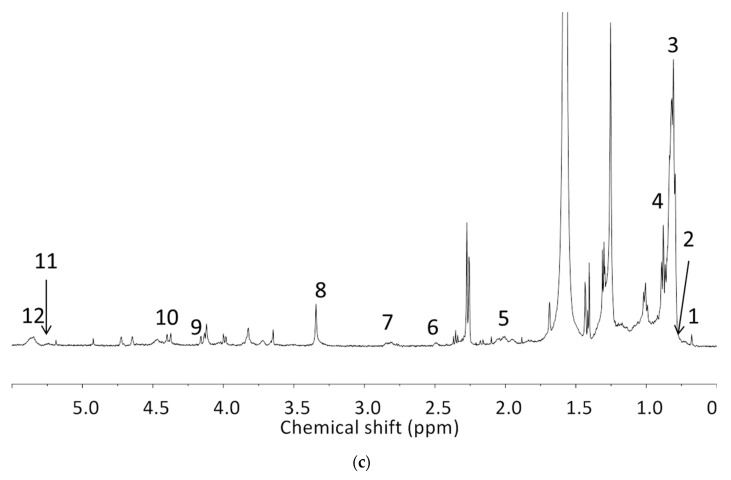
Representative ^1^H-NMR spectra from aqueous samples (**a**) from 0.8 to 3.3 ppm, (**b**) from 4.5 to 8.5 ppm and organic extracts (**c**) from 0.5 to 5.5 ppm. The numbers correspond to signal attribution for metabolite which were quantified. See Table 1 and Table 2 for signal attribution.

The analyzed spectral allowed us to identify 32 metabolites/chemical structures, characterized by a molecular weight lower than 1000 Da. The metabolites identified within the synovial fluid with ^1^H NMR spectroscopy included amino acids, organic acids, fatty acids or sugars. Representative spectra from aqueous samples and organic extracts are presented in Figure 1. As depicted in Table 1 and Table 2, the name, the chemical structure, the chemical shift, the integrated signal, the number of ^1^H and the human metabolome database (HMDB) reference are presented for each annotated metabolite. All the raw data have been registered in MetaboLights database (ref MTBLS1894).

### 2.2. Effect of Local Cryotherapy on Metabolite Concentrations

The analysis was conducted on SF samples taken before (D0) and after (D1) the local cryotherapy treatment. A total of 32 signals were analyzed with ^1^H NMR spectra. Five metabolites were significantly different between D0 and D1 (*p* < 0.05).

**Figure 2:**
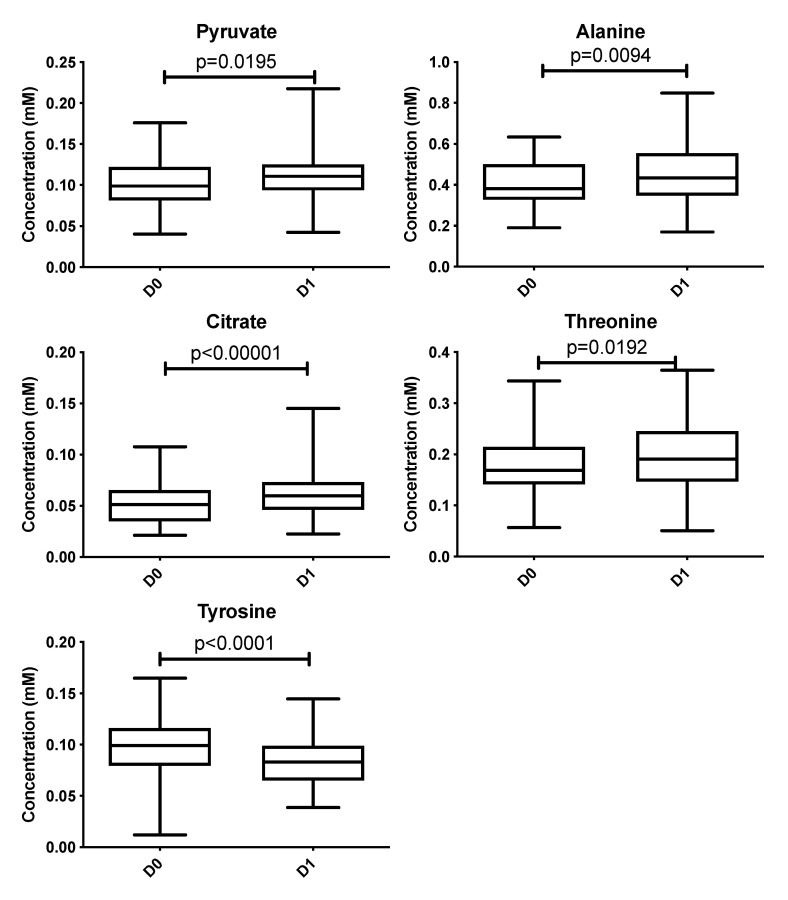
Boxplots of metabolites identified from multivariate analysis as significantly different between D0 vs. D1 at an adjusted *p* with a false discovery rate of 10%. Y-axis represents metabolite concentration (mM).

These metabolites are pyruvate, alanine, citrate, threonine and tyrosine. These metabolites’ concentrations (Figure 2, Table 3 and Table 4) were significantly higher at D1 vs D0 (*p* < 0.05) except tyrosine concentration which was significantly lower at D1 compared with D0 (*p* < 0.001). Polyunsaturated fatty acid concentration increases after local cryotherapy application, but the changes did not reach statistical significance.

For PCA and PLS-DA, the score and the loading plots were provided as Supplementary data with detailed methods.

Regarding the gender and the cooling technique effects, we did not observe any difference in metabolite concentrations between D0 and D1 (Table 5 and Table 6).

### 2.3. Pathway Analysis

**Figure 3:**
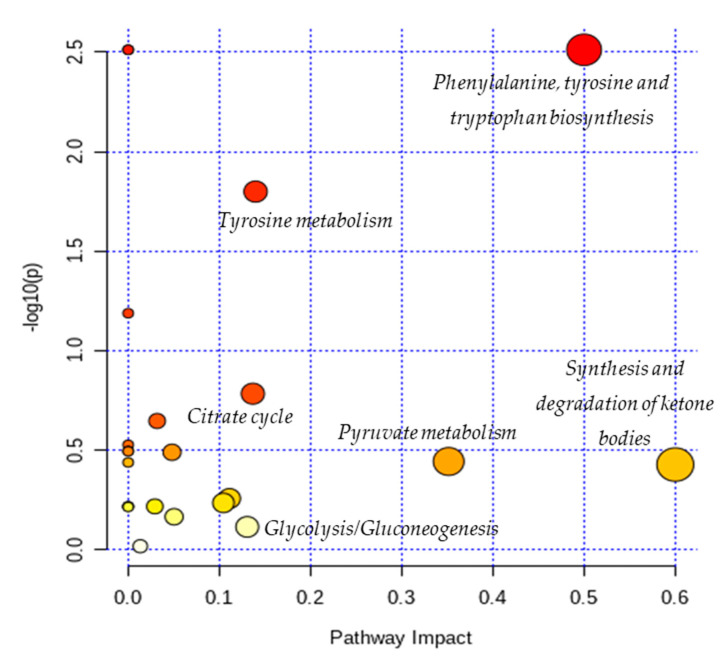
Overview of pathway analysis arranged according to the scores from enrichment analysis (y axis) and from topology analysis (x axis). The color and size of a circle (pathways) are based on its *p*-value and pathway impact value, respectively. The circles located in the top right diagonal region represents pathways with significant metabolite changes and higher impact. The pathways names with highest impact values (biggest circles) are mentioned in the figure (Phenylalanine, tyrosine and tryptophan biosynthesis; Tyrosine metabolism; Citrate cycle; Glycolysis/Gluconeogenesis; Pyruvate metabolism; Synthesis and degradation of ketone bodies).

The results from pathway analysis are presented in a graphical output (Figure 3) and a table (Table 7) which displays all the matched metabolic pathways. The 20 identified metabolites correlated with 23 metabolic pathways.

As depicted in Table 7, the topology analysis showed that five pathways (Cysteine and methionine metabolism; Butanoate metabolism; Glycolysis/Gluconeogenesis; Citrate cycle (TCA cycle); Tyrosine metabolism) have an impact value between 0.1 and 0.2, and three pathways (Pyruvate metabolism; Phenylalanine, tyrosine and tryptophan biosynthesis; Synthesis and degradation of ketone bodies) have an impact value higher than 0.2. Among them, two pathways (Phenylalanine, tyrosine and tryptophan biosynthesis; Tyrosine metabolism) have a low *p*-values according to the enrichment analysis.

## 3. Discussion

The present study aimed to identify the metabolite changes in synovial fluid taken from patients suffering from knee arthritis before and after two applications of local cryotherapy. The synovial specimens we analyzed were aliquots of the specimens obtained from the patients investigated in the study of Guillot et al. [6], where the level of inflammation was shown to decrease after cold treatment. We observed that local cryotherapy applied twice led to a significant increase in the concentration of pyruvate, alanine, citrate, threonine and a significant decrease in tyrosine concentration in inflammatory synovial fluid. These observations indicate an increased energy metabolism as observed in the pathway analysis (Table 7 and Figure 3). However, previous studies reported that some of these metabolites might have anti-oxidative and anti-inflammatory effects [28,29,30,31,32].

It has been previously reported that pyruvate and alanine are two important metabolites involved in the anti-oxidant and anti-inflammatory responses [28,31,32]. Pyruvate is a very important biological molecule involved in a large number of biological processes, such as the tricarboxylic acid (TCA) cycle, glycolysis, and glycogenesis. Besides its role in energy metabolism, this glucose metabolite is known as an effective scavenger for ROS by protecting cells against the toxic action of hydrogen peroxide (H_2_O_2_), suggesting thereby its potent antioxidant action [28,33,34]. Furthermore, pyruvate has been reported as endogenous anti-inflammatory molecules by suppressing nuclear factor (NF)-a secretion, nuclear factor-kappa (NF-k)-B expression and is able to enhance the production of anti-inflammatory cytokines [28]. Other investigators reported that ethyl pyruvate, a derivative of pyruvate, attenuated synovial inflammation and bone destruction in mice with collagen-induced arthritis through suppression of IL-17 and high mobility group box 1 protein (HMGB-1). The increase in the concentration of pyruvate that we observed in the synovial fluid of these patients could partially explain the anti-inflammatory effect of local cryotherapy previously observed in this population [6]. Our results are in line with our previous findings [6], that demonstrated that local cryotherapy inhibits the pro-inflammatory cytokine and enzyme pathways in the synovial fluid of arthritic knees. Moreover, a growing body of literature [10,16] examined the use of cryotherapy to relieve rheumatic and inflammatory diseases such as rheumatoid arthritis. Cryotherapy has been used as a rehabilitation treatment in patients suffering from inflammatory arthritis due its analgesic effect [12,18] and its efficient effects in reducing inflammatory mediator’s activity such as pro-inflammatory cytokines [6], histamine levels [35] and oxidative stress [2].

Further, we have observed that local cryotherapy increases the alanine level in the synovial fluid of arthritic patients. The raised level of alanine may be related to the glucose-alanine cycle, which consists in converting pyruvate to alanine by glutamate-pyruvate transaminase (GPT). Alanine can then be transported to the liver to act as a substrate in the gluconeogenesis process [36]. Beta-alanine is an amino acid directly involved in increasing the intramuscular carnosine content. The antioxidant capacity and the buffering effect of this compound are well documented [37,38,39,40]. Ponist et al. [37] evaluated the therapeutic aspect of carnosine in rats with arthritis. They demonstrated a systemic anti-inflammatory activity and a protective effect of carnosine against the damage caused by oxidative stress on chondrocytes. These cells are involved in preventing the breakdown of cartilage in arthritic joints. Furthermore, β-alanine supplementation has been shown to be effective in delaying neuromuscular fatigue and improving the intramuscular pH stability by reducing acidosis during physical exercise [32,39,41]. The antioxidant action of alanine has also been reported in endothelial cells through an enhanced expression of the antioxidant defense proteins Heme oxygenase (HO)-1 and ferritin [32]. Taken together, the increased level of alanine can partially explain the anti-oxidant effect induced by cold exposure in knee joint.

Moreover, the NMR analysis showed an increased citrate level in synovial fluid following the cold exposure. Despite the lack of a direct role for citrate in inflammation, it has been suggested as an important metabolite in immunity and inflammation [42]. Citrate transportation has been demonstrated to regulate the inflammatory response due to the mitochondrial citrate carrier (CIC). The latter is responsible for the transport of citrate from the mitochondria, where it is produced as part of the TCA cycle, to the cytosol. Citrate is cleaved then by citrate lyase into acetyl-CoA and oxaloacetate, precursors of nitric oxide (NO), ROS, and arachidonic acid. The CIC expression has been demonstrated to increase in the lipopolysaccharide (LPS)-activated immune cells. Moreover, CIC silencing and the CIC activity inhibition have been shown to impair the production of ROS, NO, and prostaglandins (PGs) [43]. The increased level of citrate that we observed may explain an inhibited inflammatory process by a lower degradation of citrate to acetyl-CoA and a lower inflammatory mediator expression (ROS, NO and PGs).

In addition, NMR analysis of SF samples from aqueous phase revealed a decreased concentration of tyrosine after two applications of local cryotherapy. In autoimmune diseases such as rheumatoid arthritis, pro-inflammatory mediators have been shown to activate a series of intracellular signaling pathways such as tyrosine phosphorylation [44]. This specific signaling pathway is regulated by the protein tyrosine kinase [45]. The increased level of this enzyme in SF of patients with rheumatoid arthritis was considered as an indicator of the pathogenesis, the inflammatory process and the disease state [46]. A large number of tyrosine kinase inhibitors (ex: imatinib and dasatinib) have been developed to provide therapeutic benefits in the treatment of inflammatory arthritis [47,48]. Our observations revealed that local cryotherapy reduce the tyrosine concentration, suggesting a possible attenuated inflammation response in SF after exposure to cold stimulus.

We have also observed an increased level of threonine after local cryotherapy exposure in arthritic knee. Threonine is a very important amino acid for the production and stabilization of collagen [49]. It interferes with glycine and serine to strengthen connective tissues and muscles and help them to remain elastic throughout the body [50]. Hence, the increased threonine level in synovial fluid after local cryotherapy application may accelerate the healing and the recovery from joint inflammation.

NMR analysis of organic extracts shows that local cryotherapy tends to increase polyunsaturated fatty acid (PUFA) concentration in synovial fluid. The multiples comparisons did not show a significant increase. A specific investigation of this molecule could be of interest in a future experiment with more volunteers to explore the metabolic pathways used to generate the positive effects of cryotherapy on synovial inflammation. PUFA is characterized by the presence of two or more double bonds. Its anti-inflammatory action in inflammatory diseases is well documented [51,52,53,54,55,56]. The PUFA dietary supplementation, especially by n-3 fatty acid eicosapentaenoic acid (EPA), has also been shown to inhibit the production of arachidonic acid-derived pro-inflammatory eicosanoids (prostaglandin E2 and leukotriene B4), proinflammatory cytokines, reactive oxygen species, lymphocytes reactivity [53,55,57] and to increase anti-inflammatory cytokines (e.g., IL-10) [58]. Moreover, a previous work has demonstrated that PUFA (docosahexaenoic acid) supplementation in mice with rheumatoid arthritis reduces cartilage destruction, bone damage, pro-inflammatory cytokines levels, and anti-collagen antibodies [59]. Therefore, several studies [51,53,54,57] and meta-analysis [60,61] suggested the use of PUFA as an attractive adjunctive treatment for inflammatory diseases, such as rheumatoid arthritis due to its potent capacity in reducing disease activity, NSAIDs consumption, joint pain intensity and duration of morning stiffness. In addition to its anti-inflammatory effect, the high level of PUFA in the synovial fluid may indicate a decrease in oxidative stress [62,63].

In addition, the pathway analysis showed that the metabolites with higher impact values (pyruvate, alanine, citrate, tyrosine and threonine) are involved in energy metabolic pathways such as: glycolysis/gluconeogenesis; pyruvate metabolism; citrate cycle (TCA cycle); tyrosine metabolism; Phenylalanine, tyrosine and tryptophan biosynthesis and synthesis and degradation of ketone bodies. Indeed, we suggest that local cryotherapy stimulates these metabolic pathways in patients with joint inflammation and that is may be involved in the reduced inflammatory reaction as observed in Guillot’s study [6] on this population.

This work presents nevertheless some limitations. The heterogeneous nature (gender, age) of the arthritic patients’ cohort reveals an inter-individual variability which strongly compromised the use of principal component analysis (PCA) for metabolomics approach. With using PCA, we were not able to segregate treated (cooling techniques: D1) and non-treated (without cooling: D0) samples with a high degree of accuracy. Furthermore, we had a rather low number of included individuals. Hence, we have presented only multivariate analyses in order to test the effect of cryotherapy treatment on the evolution of each metabolite. A further limitation of this study was that we were unable to include control SF samples from healthy individuals and from patients. It is always complicated to obtain SF samples from healthy subjects and from patients as joint aspirations (two in our study design) remain invasive and painful procedures.

In our sample preparation, the choice of using TSP as a reference for quantification could be questioned. Indeed, it is known that TSP could bind proteins in biofluids containing proteins such as SF. Beckonert et al. recommend to determine absolute concentrations using an internal standard of known concentration, as formate, or with addition into the sample of a standard of the analyte of interest [64]. However, before the addition of TSP, SF underwent treatment procedure with a filtration step with 10K centrifugal device to remove proteins. We have also checked that spectra did not show any presence of proteins. Finally, it has to be noted that the concentration of glucose, lactate, citrate and pyruvate in SF reported in the scientific literature using various techniques including HPLC and enzymatic assays in human and animal models are in accordance with our data [65,66,67].

## 4. Experimental Methods

### 4.1. Patients

The included patients (*n* = 46, Age: 60 ± 14 years, 22 men and 24 women) were suffering from non-septic knee arthritis. They were enrolled in the investigation, which was declared and approved by the local ethics committee (clinicaltrials.gov: NCT03850392, Comité de Protection des Personnes–Est II: 12-664). The patients were included consecutively after signed informed consent.The patients received no biological or conventional DMARD treatment for 6 months before inclusion. They did not receive any local cryotherapy in the month before inclusion.

### 4.2. Study Design and Patient Sample Collection

The patients were then randomized to receive either local ice (Thermogel^®^, Artsana, Grandate, Italy; 30-min application; *n* = 30) or hyperbaric cold CO_2_ at −78 °C (Cryo+^®^, Cryonic, Salins-les-Bains, France; 2-min application; *n* = 16). They received two applications of cooling technique (ice or cold CO_2_) at an 8-h interval (9 a.m. and 5 p.m.). Synovial fluid (SF) samples were taken from the knee joint on D0 (just before the first application of cold) then D1 (24 h later) at the same time of the day in order to limit the risk of bias linked to circadian variations. The SF specimens collected in syringe were transferred to Falcon tubes (15 mL) and centrifuged twice at 2500× *g* at 4 °C during 15 min. Supernatants were then frozen at −80 °C into 500 µL Eppendorf tubes. No blood contamination was observed.

### 4.3. Sample Preparation for NMR Analysis

Solvents were purchased from Sigma Aldrich^®^, Saint-Quentin Fallavier, France and NMR tubes from CortecNet^®^, Voisins-Le-Bretonneux, France. Sample preparation was conducted on 46 patients for aqueous analysis and 44 patients for organic extracts because of low volumes of SF for 2 patients.

Organic extracts were prepared using a biphasic extraction [68,69]. A total of 400 µL of SF samples was thawed at room temperature and mixed with 450 µL of cold methanol and 450 µL of cold chloroform. Samples were vortexed 30 s after each step and were placed into ice for twenty minutes. Samples were centrifugated at 8000× *g* at 4 °C during 10 min. A total of 400 µL of organic fraction was placed in a 1.5 mL eppendorf tube and evaporated under a nitrogen flow. Dry pellets were stored at −20 °C until NMR analysis. Before NMR analysis, pellets were suspended in 600 µL of a mixture of 90% chloroform-D and 10% chloroform-D containing 0.05% (*v*/*v*) of Tetramethylsilane (TMS). A total of 500 µL was placed in a 5 mm pyrex NMR sample tube.

Aqueous samples were prepared by filtration to remove proteins. SF samples were thawed at room temperature. For each sample, two centrifugal devices (Pall Nanosep^®^ centrifugal device (from Merck KGaA, Darmstadt, Germany) with Omega membrane 10 K from Sigma Aldrich in order to remove protein) were washed twice by addition of 500 µL of distilled water by centrifugation at 8000× *g* at room temperature during 10 min. 200 µL of SF sample was then mixed with 400 µL of deuterium oxide. Diluted samples were centrifuged at 9000× *g* at room temperature during 25 min, using the first centrifugal device. Non filtrated fraction was put on the second centrifugal devices and were centrifuged at 9000× *g* at room temperature during 25 min. A total of 250 µL of pooled filtrate was mixed with 250 µL of deuterium oxide and 25 µL of deuterium oxide containing 0.05 wt. % 3-(trimethylsilyl)propionic-2,2,3,3-d4 acid, sodium salt. pH was adjusted at 2.50 ± 0.05 with addition of concentrated chlorhydric acid. A total of 500 µL was introduced in a 5 mm pyrex NMR sample tube. NMR analysis occurred immediately after the preparation.

### 4.4. Proton NMR Spectroscopy

All NMR experiments and data analysis were performed randomly, on one unique period for each type of samples.

(a) Instrument description: Spectra were obtained with a 11.75 Tesla spectrometer 500SB Bruker (Bruker BioSpin^®^, Wissenbourg, France)—spectrometer consoles were Avance I and Neo for organic extracts and aqueous samples, respectively. The magnet was equipped with a 5 mm broad band inverse (BBi) ^1^H/^13^C probe (Bruker BioSpin^®^, Wissenbourg, France). Tuning and matching were automated. Automated gradient shimming for Z coils were used and manual optimization were performed when needed. Spectrometer was controlled with TopSpin 4.0.8 software (Bruker BioSpin^®^, Wissenbourg, France).
(b) Spectra acquisition parameters: Organic extracts were thermostated at 293 K without spinning. One-dimensional (1-D) ^1^H NMR spectra were obtained at 500.09 MHz using a 1-D experiment impulsion acquisition sequence (zg). Spectra were obtained in 9 min by accumulating 64 free induction decay (FID) and 4 dummy scans. Acquisition time was 5.45 s with a spectral width of 6 kHz collected in 64 K data points and an additional relaxation delay d1 of 2 s. The 90° pulse delay was 6.7 µs with power level at −1 dB. Receiver gain was set at 512 for each sample. Aqueous samples were thermostated at 293 K without spinning. One-dimensional (1-D) ^1^H NMR spectra were obtained at 500.09 MHz using a 1-D experiment impulsion acquisition sequence using a noesygppr1d pulse sequence (Bruker) with presaturation delay during relaxation delay (d1 = 5 s) and mixing time (d8 = 10 ms). Homospoil/gradient pulse p16 was 1 ms and delay for homospoil/gradient recovery was 200 µs. Spectra were obtained in 23 min and 20 s by accumulating 128 free induction decay (FID) and 4 dummy scans. Acquisition time was 5.57 s with a spectral width of 6 kHz collected in 64 K data points. The 90° pulse delay was 8 µs with power level at −11.78 dB. In addition, the residual water resonance was pre-saturated with a 44.19 dB field strength irradiation. Receiver gain was set at 32 for each sample.
(c) Spectra processing parameters: Raw data were registered in MetaboLights database [70]. All spectra were processed with TopSpin 4.0.8 software (Bruker BioSpin^®^, Wissenbourg, France). Typical processing parameters were application of Fourier transform without line broadening or zero filling. Chemical shifts (δ in ppm) were reported relative to the signal of the TSP or TMS at 0 ppm. Phase correction and baseline correction were corrected manually when needed. Isolated signal integrations were performed relative to reference signal (TSP or TMS).

For aqueous samples, TSP peak was also used as reference for quantification. Calibration of TPS peak, which had the same intensity in all spectra, was established from a 3 mM creatine solution containing TPS. The concentrations in the NMR tube of each metabolite were calculated in mmoles by liter and formulae for concentration calculations are presented below:

(1) $$\left[\mathrm{TSP}\right]=\frac{\int \mathrm{TSP}}{\int \mathrm{Creatine}}\times \frac{n_{H_{\mathrm{Creatine}}}}{n_{H_{\mathrm{TSP}}}}\times \left[\mathrm{Creatine}\right]$$

(2) $$\left[\mathrm{Metabolite}\right]=\frac{\int \mathrm{Metabolite}}{\int \mathrm{TSP}}\times \frac{n_{H_{\mathrm{Creatine}}}}{n_{H_{\mathrm{Metabolite}}}}\times \left[\mathrm{TSP}\right]$$

Metabolite concentrations in the SF were then corrected according to dilution factors as presented in the sample preparation section. A comparison of two options of quantification is presented in Supplementary data; it concerns the use of TSP with the SF or in a coaxial NMR tube.

For organic extracts, signals were integrated relative to TMS signal which remained the same in all spectra. Integrated signals corresponding to a chemical function/pattern with a group of metabolites were studied. No absolute quantitation was performed.

Annotation of the metabolites was performed using the Human Metabolome Database [71], the Magnetic Resonance Metabolomics Database [72], our own database performed at pH = 2.50 ± 0.05, and literature on biofluids [73,74]. According to the Metabolite Identification Task Group from the Metabolomics Standards Initiative, the level of identification was 2 for aqueous samples and 3 for organic phase [75].

### 4.5. Data Processing and Statistical Analysis

#### 4.5.1. Multivariate Analysis

Statistical analyses were performed using specialized statistical software (https://www.graphpad.com/). The Gaussian distribution was tested for each variable using the Shapiro–Wilk test. Multiples comparisons were used to assess the significant difference between metabolite concentrations in synovial fluid of treated arthritic knees (D1) and non-treated arthritic knees (D0) and *p*-values were adjusted using a false discovery rate (FDR) procedure and a desire FDR set at 10% according to adaptative method of Benjamini, Krieger and Yekutieli with a desire FDR (Q) at 10%. Gender and the technique impacts were also tested for metabolites that presented a significant difference in their concentrations between D0 and D1. The results of the tests were considered significant at *p* ≤ 0.05 (adjusted *p* with an FDR of 10%). The results were expressed as mean and standard deviation (SD).

The principal component analysis (PCA) and partial least squares-discriminant analysis (PLS-DA) methods and results were provided in the Supplementary data.

#### 4.5.2. Pathway Analysis

We have performed a pathway analysis using Metaboanalyst 4.0 (https://www.metaboanalyst.ca), an easy-to-use web-based tool suite for comprehensive and integrative metabolomics data analysis [76]. The metabolites’ concentrations table was normalized by a pooled average sample from control group (Day-0) and auto-scaled. We have used KEGG library for “*Homo sapiens*” and we have launched two forms of pathway analysis: enrichment (global test) and topology (relative-betweenness centrality) to assess the *p*-values and the pathway impact values respectively.

## 5. Conclusions

Overall, our observation may explain in part the reduced joint inflammation in synovial fluid following local cooling application reported in the study of Guillot et al. [6] on this population. As observed in the literature, our results suggest that the anti-inflammatory and the antioxidant role of cryotherapy could partially be explained by the increased level of metabolites involved in the energy metabolism: glucose metabolite (pyruvate), organic acid (citrate), amino acids (alanine, tyrosine and threonine) and polyunsaturated fatty acid. However, more specific studies for each metabolite are of utmost importance to investigate the effect of cryotherapy on molecular pathways and on the activity of these metabolites in arthritic synovial fluid. It would also be of great interest to develop investigations where the clinical relevance of using cryotherapy on regular basis in arthritic patients could be highlighted.

## Figures and Tables

**Table 1:** Names, peak numbering on spectra, HMBD ID, chemical structure and chemical shift assignments of the metabolites observed in the NMR spectra of Synovial Fluid samples for the aqueous phase.

Names	Peak Numbering on Spectra (Figure 1a,b)	HMBD ID	Chemical Structure	Chemical Shift for Signal Used (ppm); Multiplicity; Hydrogen Number
Valine	1	HMDB0000883	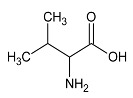	1.047; d; 3
Ethanol	2	HMDB0000108	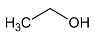	1.170; t; 3
3-hydroxybutyrate	3	HMDB0000357	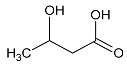	1.235; d; 3
Threonine	4	HMDB0000167	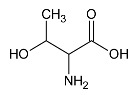	1.329; d; 3
Lactate	5	HMDB0000190	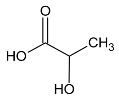	1.409; d; 3
Alanine	6	HMDB0000161	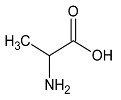	1.514; d; 3
n-butyrate	7	HMDB0000039	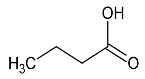	1.565; m; 2
Acetate	8	HMDB0000042	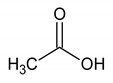	1.798; s; 3
N-acetylaspartyl glutamic acid	9	HMDB0001067	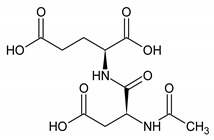	2.078; s; 3
Methionine	10	HMDB0000696	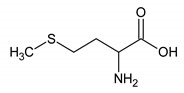	2.121; s; 3
Acetone	11	HMDB0001659	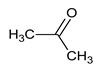	2.216; s; 6
Acetoacetate	12	HMDB0000060	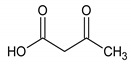	2.303; s; 3
Pyruvate	13	HMDB0000243	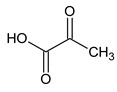	2.384; s; 3
Citrate	14	HMDB0000094	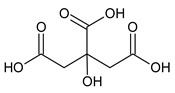	2.826; syst AB; 2 2.859; syst AB; 2
Creatinine	15	HMDB0000562	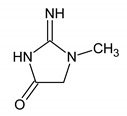	3.055; s; 3
Dimethylsulfone	16	HMDB0004983	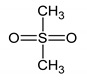	3.127; s; 6
Ethanolamine	17	HMDB0000149	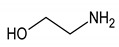	3.140; t; 2
Betaine	18	HMDB0000043	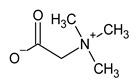	3.267; s; 9
β-glucose	19	HMDB0000516	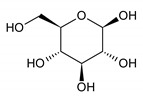	4.633; d; 1
α-glucose	20	HMDB0003345	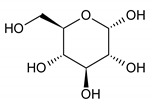	5.217; d; 1
Tyrosine	21	HMDB0000158	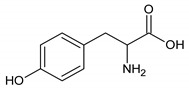	6.889; d; 2 7.189; d; 2

**Table 2:** Names, peak numbering on spectra, chemical structure and chemical shift assignments of the metabolites observed in the NMR spectra of synovial fluid samples for organic phase.

Attribution	Peak Numbering on Spectra (Figure 1b)	Chemical Structure of Molecule or Group	Chemical Shift for Signal Used (In ppm); Multiplicity; Number of Hydrogens
Cholesterol HMDB0000067	1	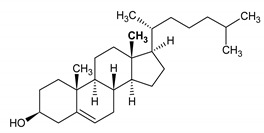	0.679; s; 3
Methyl group for fatty acyl chain with ω6 double bound	2	CH_3_-(CH_2_)_4_-CH=	0.808; t; 3
Methyl group for fatty acyl chain	3	CH_3_-(CH_2_)_n_-CH_2_-COOR	0.887; t; 3
Methyl group for fatty acyl chain with ω3 double bound	4	CH_3_-CH_2_-CH=	1.008; t; 3
Methylene group next to double bound	5	-CH_2_-CH=	1.996; m; 2
Methylene group next to carbonyl	6	-CH_2_-COOR	2.255; m; 2
Methylene group between two double bound	7	-CH_2_-CH=CH-	2.811; m; 2
Choline compounds	8	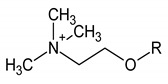	3.318; s; 9
Methylene groups of glycerol body	9	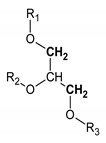	4.123 and 4.166; AB syst; 2 4.372 and 4.398; AB syst; 2
Methine group of glycerol body	10	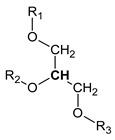	5.188; m; 1
Hydrogen bounded to a carbon involved in a double bond	11	-CH=CH-	5.368; m; 1

**Table 3:** Concentration (mM) of metabolites in aqueous samples before (D0) and after (D1) the local cryotherapy treatment (*n* = 46) (results are expressed as mean ± SD and adjusted *p* value; bolding indicates significant changes in the metabolite concentration with a false discovery rate of 10%).

Names	Mean ± SD		Adjusted *p* Value
	D0	D1	
**Valine**	0.32 ± 0.11	0.33 ± 0.10	0.17
Ethanol	0.41 ± 0.40	0.47 ± 0.74	0.47
3-hydroxybutyrate	0.20 ± 0.30	0.16 ± 0.15	0.67
**Threonine**	0.18 ± 0.06	0.19 ± 0.06	**0.02**
Lactate	4.40 ± 2.23	4.06 ± 1.72	0.12
**Alanine**	0.40 ± 0.12	0.44 ± 0.14	**0.01**
n-butyrate	0.11 ± 0.04	0.12 ± 0.04	0.28
Acetate	0.02 ± 0.01	0.02 ± 0.01	0.22
NAAG	0.07 ± 0.03	0.07 ± 0.03	0.54
Methionine	0.05 ± 0.02	0.06 ± 0.02	0.27
Acetone	0.05 ± 0.10	0.06 ± 0.13	0.58
Acetoacetate	0.10 ± 0.11	0.09 ± 0.07	0.21
**Pyruvate**	0.10 ± 0.03	0.12 ± 0.04	**0.02**
**Citrate**	0.05 ± 0.02	0.06 ± 0.02	**<0.01**
Creatinine	0.07 ± 0.04	0.08 ± 0.04	0.09
Dimethylsulfone	0.04 ± 0.02	0.05 ± 0.02	0.40
Ethanolamine	0.059 ± 0.07	0.06 ± 0.05	0.25
Betaine	0.05 ± 0.02	0.05 ± 0.02	0.95
β-glucose	3.41 ± 1.65	3.57 ± 1.84	0.17
α-glucose	2.62 ± 1.25	2.71 ± 1.33	0.17
**Tyrosine**	0.10 ± 0.03	0.08 ± 0.03	**<0.01**

**Table 4:** Integration of signal (relative to TMS integrate signal) in organic extract before (D0) and after (D1) the local cryotherapy treatment (*n* = 44) (Mean ± SD; adjusted *p* value with a false discovery rate of 10%).

Names	Mean ± SD		Adjusted *p* Value
	D0	D1	
Cholesterol	3.01 ± 3.68	2.57 ± 2.06	0.60
Methyl group for fatty acyl chain with ω6 double bound	264.80 ± 369.90	224.20 ± 213.80	0.60
Methyl group for fatty acyl chain	80.73 ± 101.30	72.97 ± 60.86	0.72
Methyl group for fatty acyl chain with ω3 double bound	45.72 ± 60.04	38.84 ± 38.58	0.70
Methylene group next to double bound	16.89 ± 16.38	17.14 ± 15.23	0.76
Methylene group next to carbonyl	12.81 ± 3.84	13.37 ± 5.33	0.58
Methylene group between two double bound	4.23 ± 4.47	5.92 ± 5.13	0.04
Choline compounds	7.11 ± 5.08	7.89 ± 5.50	0.45
Methylene groups of glycerol body	1.93 ± 0.97	2.15 ± 1.03	0.22
Methine group of glycerol body	1.21 ± 1.91	1.00 ± 1.45	0.86
Hydrogen bounded to a carbon involved in a double bond	11.76 ± 7.35	12.28 ± 7.28	0.67

**Table 5:** Difference between concentration (mM) of metabolites in aqueous samples measured at D1 and D0 in men and women (Mean ± SD; adjusted *p* value with a false discovery rate of 10%) and in patient receiving either local ice or hyperbaric cold CO_2_ (Mean ± SD; adjusted *p* value with a false discovery rate of 10%).

Names	Mean ± SD		Adjusted *p* Value	Mean ± SD		Adjusted *p* Value
	Men (*n* = 22)	Women (*n* = 24)		Local Ice (*n* = 30)	CO_2_ (*n* = 16)	
Valine	0.006 ± 0.055	0.018 ± 0.062	0.478	0.018 ± 0.062	0.002 ± 0.050	0.355
Ethanol	0.035 ± 0.760	0.081 ± 0.437	0.183	0.171 ± 0.700	−0.151 ± 0.295	0.133
3-hydroxybutyrate	−0.002 ± 0.146	−0.069 ± 0.379	0.752	−0.043 ± 0.344	−0.025 ± 0.156	0.354
Threonine	0.010 ± 0.030	0.014 ± 0.037	0.677	0.013 ± 0.038	0.011 ± 0.027	0.861
Lactate	−0.501 ± 1.821	−0.189 ± 0.860	0.819	−0.245 ± 0.898	−0.513 ± 2.059	>0.999
Alanine	0.029 ± 0.093	0.050 ± 0.107	0.703	0.053 ± 0.094	0.016 ± 0.110	0.086
n-butyrate	0.014 ± 0.032	−0.001 ± 0.033	0.137	0.007 ± 0.031	0.004 ± 0.038	0.738
Acetate	0.002 ± 0.016	0.003 ± 0.009	0.649	0.004 ± 0.014	0.000 ± 0.011	0.363
NAAG	−0.005 ± 0.026	0.007 ± 0.017	0.036	0.003 ± 0.020	−0.002 ± 0.019	0.392
Methionine	0.002 ± 0.022	0.005 ± 0.017	0.539	0.004 ± 0.021	0.004 ± 0.017	0.624
Acetone	0.021 ± 0.072	−0.007 ± 0.022	0.066	0.005 ± 0.054	0.009 ± 0.052	0.758
Acetoacetate	−0.001 ± 0.041	−0.028 ± 0.140	0.439	−0.018 ± 0.127	−0.010 ± 0.039	0.882
Pyruvate	0.011 ± 0.036	0.012 ± 0.026	0.921	0.011 ± 0.028	0.013 ± 0.035	0.803
Citrate	0.010 ± 0.010	0.007 ± 0.016	0.426	0.009 ± 0.017	0.008 ± 0.012	0.458
Creatinine	0.000 ± 0.018	0.013 ± 0.026	0.054	0.011 ± 0.025	0.001 ± 0.019	0.160
Dimethylsulfone	0.003 ± 0.027	0.003 ± 0.009	0.310	0.003 ± 0.020	0.001 ± 0.019	0.811
Ethanolamine	0.003 ± 0.067	−0.001 ± 0.019	0.424	0.002 ± 0.056	−0.002 ± 0.030	0.614
Betaine	0.001 ± 0.011	−0.001 ± 0.009	0.498	−0.001 ± 0.009	0.002 ± 0.010	0.403
β-glucose	0.286 ± 1.230	0.039 ± 1.640	0.973	0.285 ± 1.620	−0.067 ± 1.058	0.146
α-glucose	0.217 ± 0.100	−0.030 ± 1.181	0.478	0.017 ± 1.180	−0.061 ± 0.917	0.244
Tyrosine	−0.010 ± 0.0151	−0.016 ± 0.021	0.210	−0.016 ± 0.017	−0.008 ± 0.022	0.110

**Table 6:** Integration of signal (relative to TMS integrated signal) in organic extract at D1 and D0 in men and women (Mean ± SD; adjusted *p* value with a false discovery rate 10%)) and in patients receiving either local ice or hyperbaric cold CO_2_ (Mean ± SD; adjusted *p* value with a false discovery rate of 10%).

Names	Mean ± SD		Adjusted *p* Value	Mean ± SD		Adjusted *p* Value
	Men (*n* = 22)	Women (*n* = 22)		Local Ice (*n* = 29)	CO_2_ (*n* = 15)	
Cholesterol	0.16 ± 3.09	−1.03 ± 5.01	0.95	−0.81 ± 4.98	0.28 ± 1.66	0.38
Methyl group for fatty acyl chain with ω6 double bound	51.87 ± 310.90	−133.10 ± 497.30	0.45	−70.63 ± 517.70	17.45 ± 36.89	0.21
Methyl group for fatty acyl chain	21.11 ± 86.53	−36.62 ± 133.70	0.19	−13.64 ± 141.50	3.62 ± 19.80	0.70
Methyl group for fatty acyl chain with ω3 double bound	7.87	−21.62 ± 81.04	0.67	−11.28 ± 87.92	1.63 ± 14.50	0.73
Methylene group next to double bound	5.41 ± 15.64	−4.61 ± 18.36	0.09	−2.14 ± 20.48	4.61 ± 8.75	0.12
Methylene group next to carbonyl	0.35 ± 5.34	0.77 ± 5.89	0.92	−0.58 ± 5.79	2.75 ± 4.48	0.042
Methylene group between two double bound	3.68 ± 5.68	0.62 ± 5.39	0.11	1.49 ± 6.29	3.22 ± 4.18	0.33
Choline compounds	1.68 ± 5.76	−0.16 ± 6.33	0.33	−0.00 ± 6.81	2.41 ± 3.79	0.15
Methylene groups of glycerol body	0.279 ± 1.10	0.17 ± 1.26	0.76	0.17 ± 1.33	0.33 ± 0.82	0.62
Methine group of glycerol body	−0.36 ± 1.96	−0.06 ± 0.79	0.67	−0.03 ± 0.69	−0.56 ± 2.38	0.34
Hydrogen bounded to a carbon involved in a double bond	1.72 ± 8.54	−0.61 ± 8.58	0.38	0.58 ± 9.47	0.43 ± 6.77	0.95

**Table 7:** Results of pathway analysis with MetaboAnalyst 4.0 (https://www.metaboanalyst.ca) indicating the total number of the involved metabolites and those detected in our data with the *p*-value and the impact of each metabolic pathway.

	Metabolites		Pathway Analysis	
	Total Number	Detected	*p*-Value	Impact
Synthesis and degradation of ketone bodies	5	2	0.373	0.60
Phenylalanine, tyrosine and tryptophan biosynthesis	4	1	0.003	0.50
Pyruvate metabolism	22	3	0.360	0.35
Tyrosine metabolism	42	3	0.016	0.14
Citrate cycle (TCA cycle)	20	2	0.164	0.14
Glycolysis / Gluconeogenesis	26	5	0.766	0.13
Butanoate metabolism	15	3	0.552	0.11
Cysteine and methionine metabolism	33	2	0.573	0.10
Glycine, serine and threonine metabolism	33	3	0.682	0.05
Alanine, aspartate and glutamate metabolism	28	4	0.324	0.05
Glyoxylate and dicarboxylate metabolism	32	3	0.225	0.03
Galactose metabolism	27	1	0.605	0.03
Glycerophospholipid metabolism	36	1	0.958	0.01
Ubiquinone and other terpenoid-quinone biosynthesis	9	1	0.003	0.00
Phenylalanine metabolism	10	1	0.003	0.00
Aminoacyl-tRNA biosynthesis	48	5	0.064	0.00
Arginine and proline metabolism	38	1	0.297	0.00
Selenocompound metabolism	20	1	0.318	0.00
Pantothenate and CoA biosynthesis	19	1	0.320	0.00
Valine, leucine and isoleucine degradation	40	2	0.364	0.00
Fructose and mannose metabolism	20	1	0.605	0.00
Amino sugar and nucleotide sugar metabolism	37	1	0.605	0.00
Valine, leucine and isoleucine biosynthesis	8	2	0.609	0.00

## Supplementary Materials

The following are available online at https://www.mdpi.com/2218-1989/10/11/460/s1, SD1-Complementary method for multivariate analysis, Figure S1: Multivariate analysis results on aqueous samples from automatic bucketing, Figure S2: Multivariate analysis results on aqueous samples from manual integration, Figure S3: Multivariate analysis results on organic extracts from automatic bucketing, Figure S4: Multivariate analysis results on organic extracts from manual integration, Table S1: Concentration (mM) of metabolites of one SF sample calculated using two methods: reference mixed with the SF and reference in an insert.
