# Supplementary material for: 1H-NMR-Based Analysis for Exploring Knee Synovial Fluid Metabolite Changes after Local Cryotherapy in Knee Arthritis Patients
Source: Metabolites. 2020 Nov 13;10(11):460. doi: 10.3390/metabo10110460 (PMC7696760; doi:10.3390/metabo10110460)
Supplement: Supplementary file 1 [file metabolites-10-00460-s001.zip › metabolites-909405-supplementary-proof/metabolites-909405-supplementary-proof.pdf]

Supplementary data for "1H-NMR-based analysis for exploring knee synovial fluid metabolite changes after local cryotherapy in knee arthritis patients"

SD1 – Method for multivariate analysis

Multivariate analysis was performed on spectra from aqueous sample and organic extract individually. For each type of sample, two bucketing methods were conducted to generate two data matrices: (1) Automatic bucketing : bucket width 0.05 ppm, normalized to the total integral of all individual (from 0.6 to 10 ppm with exclusion of water signal from 4.4 to 5.6 ppm for aqueous samples and from 0.6 to 10 ppm with exclusion of chloroform signal from 7.0 to 8.0 ppm for organic extracts). We used Amix viewer from Bruker® to generate matrices (2) Manual bucketing with integration of isolated and identified signals related to TSP signal. We used TopSpin 4.0.8 software (Bruker BioSpin®). We used a Pareto scaling before generating the score and loading plots from PCA and PLS-DA using Simca-P 11 software from Umétrics®.

Supplementary data for "1H-NMR-based analysis for exploring knee synovial fluid metabolite changes after local cryotherapy in knee arthritis patients"

SD2 Multivariate analysis results on aqueous samples from automatic bucketing

(a) PCA score plot
(b) PCA loading plot
(c) PLS-DA score plot
(d) PLS-DA loading plot

Supplementary data for "1H-NMR-based analysis for exploring knee synovial fluid metabolite changes after local cryotherapy in knee arthritis patients"

(a)

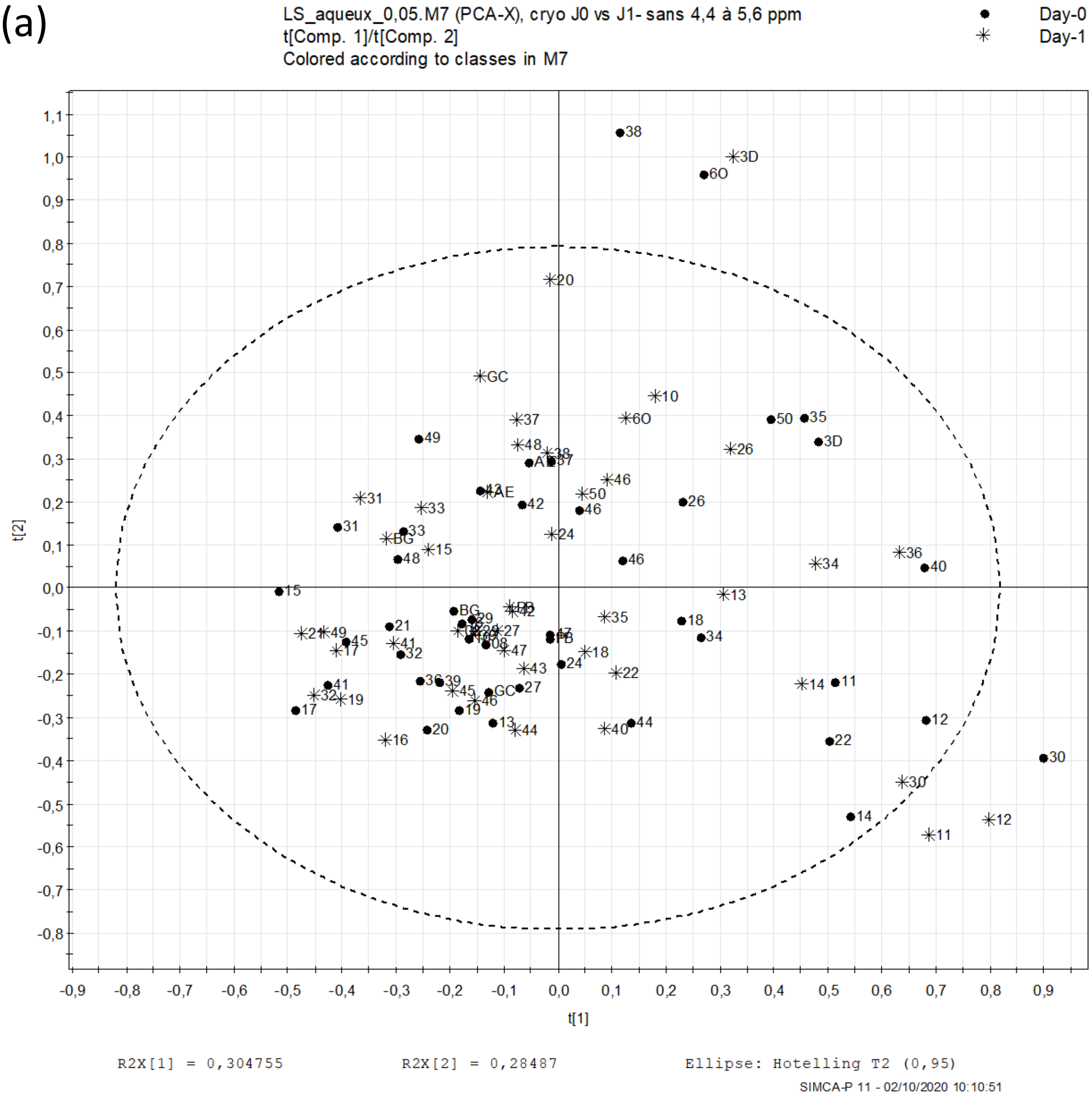

Supplementary data for "1H-NMR-based analysis for exploring knee synovial fluid metabolite changes after local cryotherapy in knee arthritis patients"

(b)

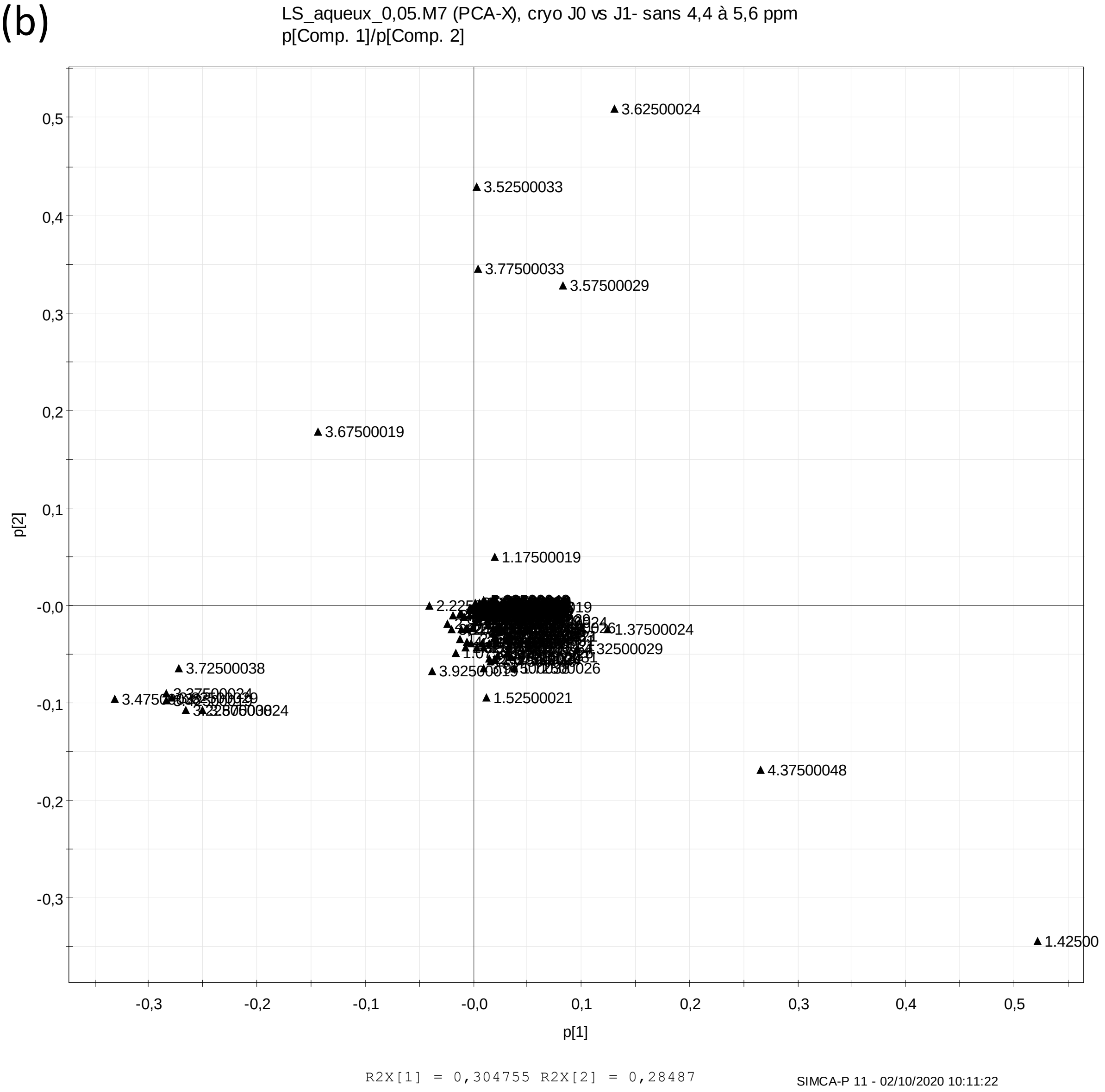

Supplementary data for “1H-NMR-based analysis for exploring knee synovial fluid metabolite changes after local cryotherapy in knee arthritis patients”

(c)

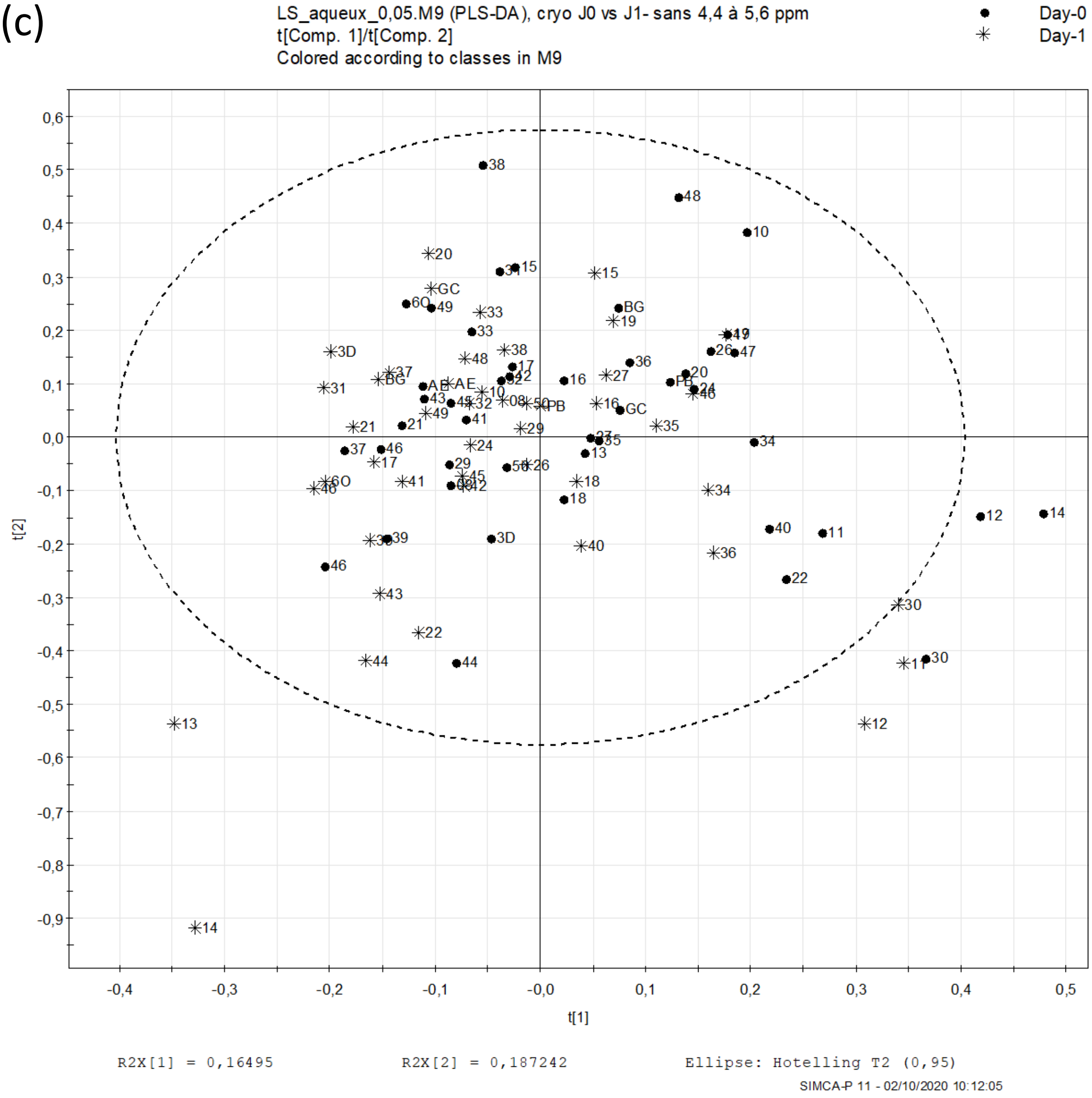

Supplementary data for "1H-NMR-based analysis for exploring knee synovial fluid metabolite changes after local cryotherapy in knee arthritis patients"

(d)

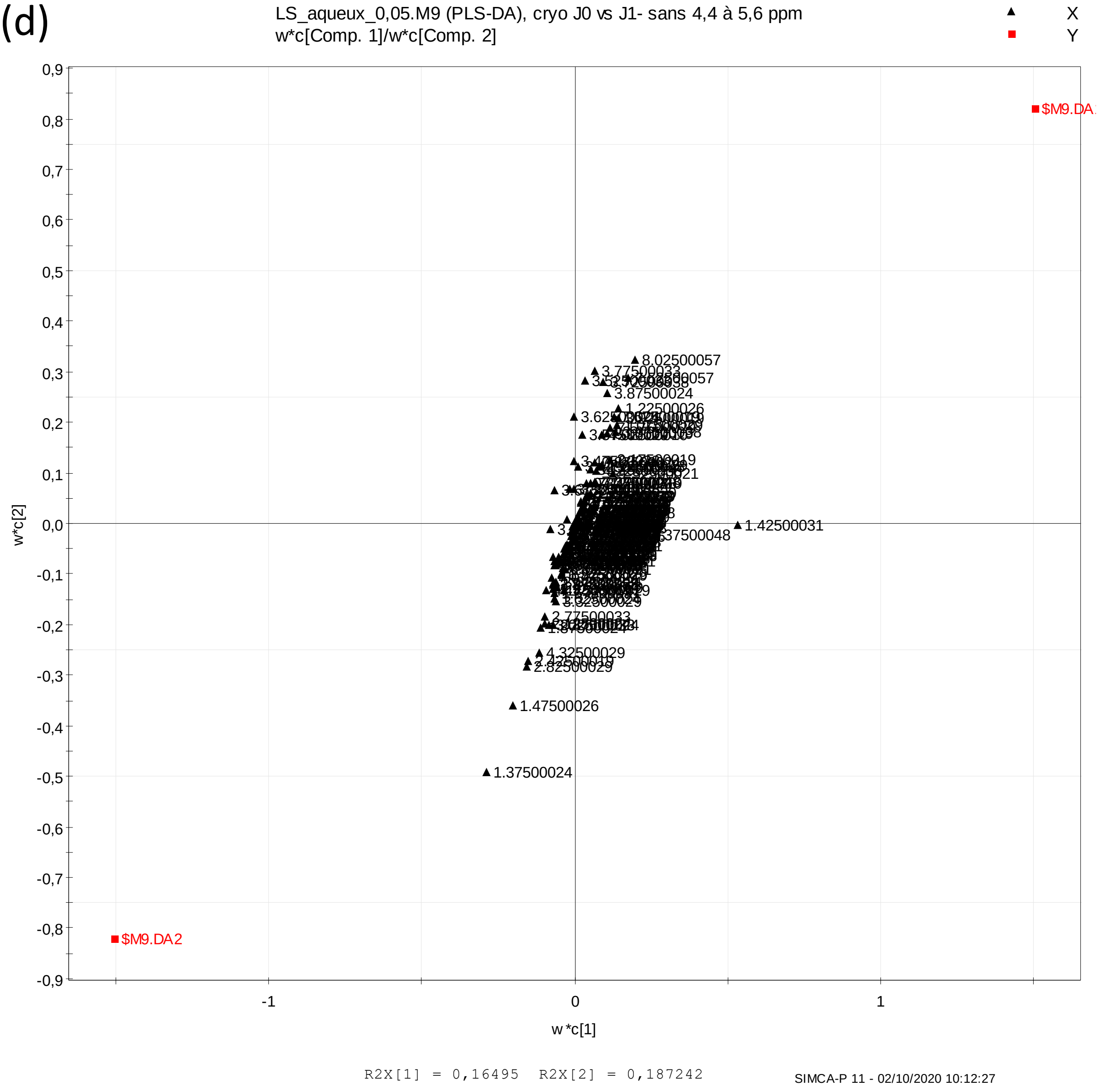

Supplementary data for "1H-NMR-based analysis for exploring knee synovial fluid metabolite changes after local cryotherapy in knee arthritis patients"

SD3 Multivariate analysis results on aqueous samples from manual integration

(a) PCA score plot
(b) PCA loading plot
(c) PLS-DA score plot
(d) PLS-DA loading plot

Supplementary data for “1H-NMR-based analysis for exploring knee synovial fluid metabolite changes after local cryotherapy in knee arthritis patients”

(a)

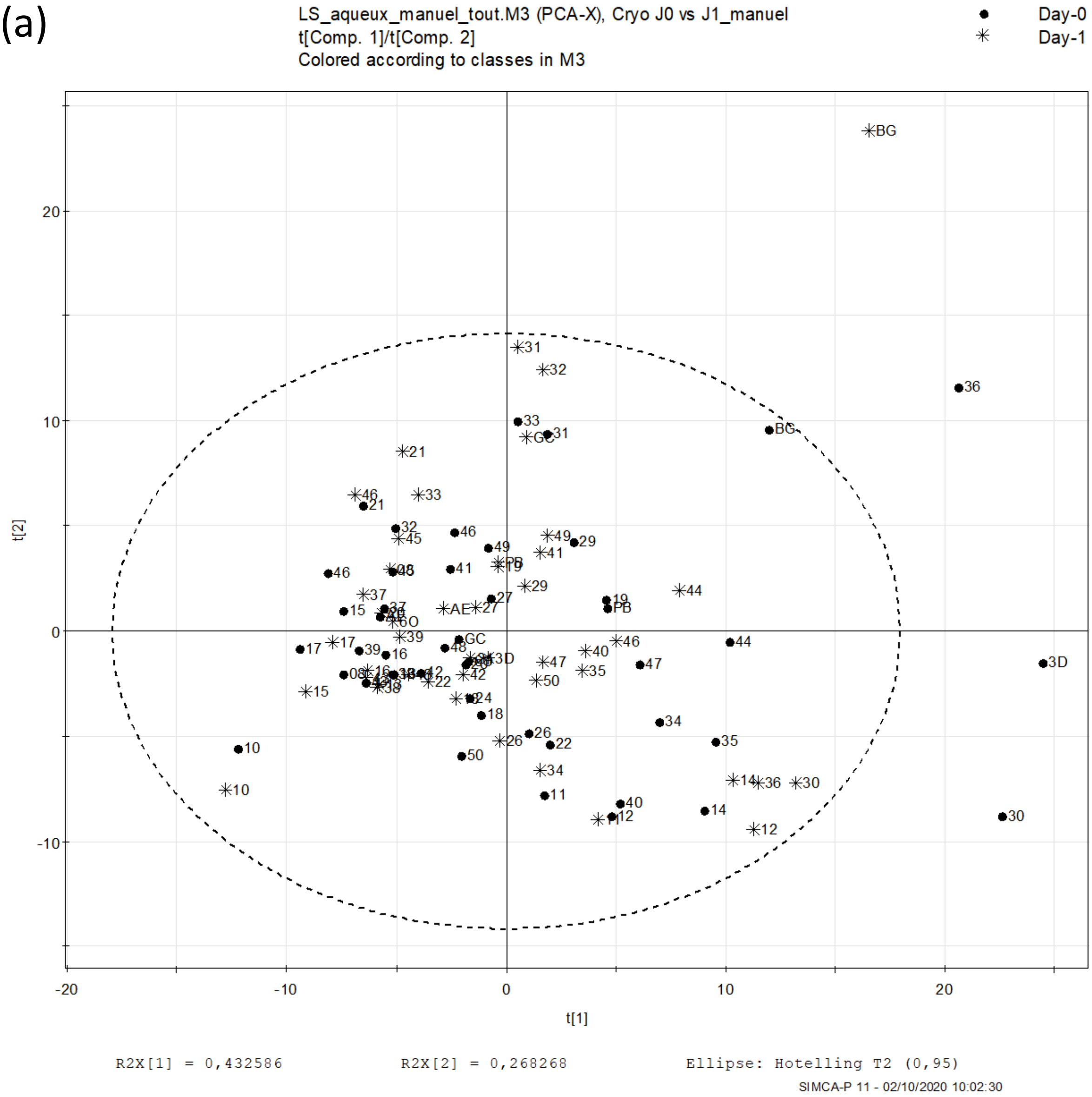

Supplementary data for "1H-NMR-based analysis for exploring knee synovial fluid metabolite changes after local cryotherapy in knee arthritis patients"

(b)

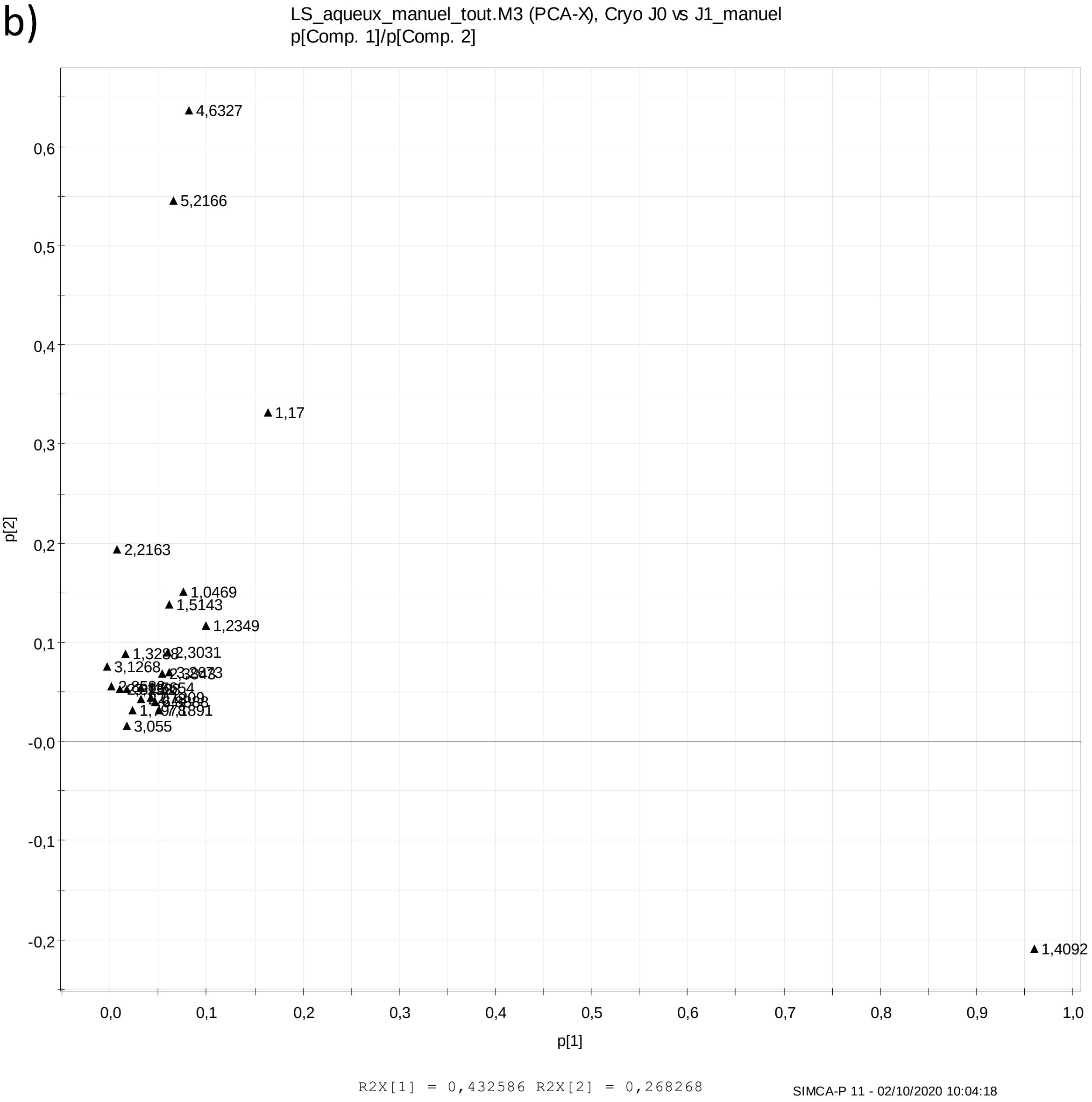

Supplementary data for "1H-NMR-based analysis for exploring knee synovial fluid metabolite changes after local cryotherapy in knee arthritis patients"

(c)

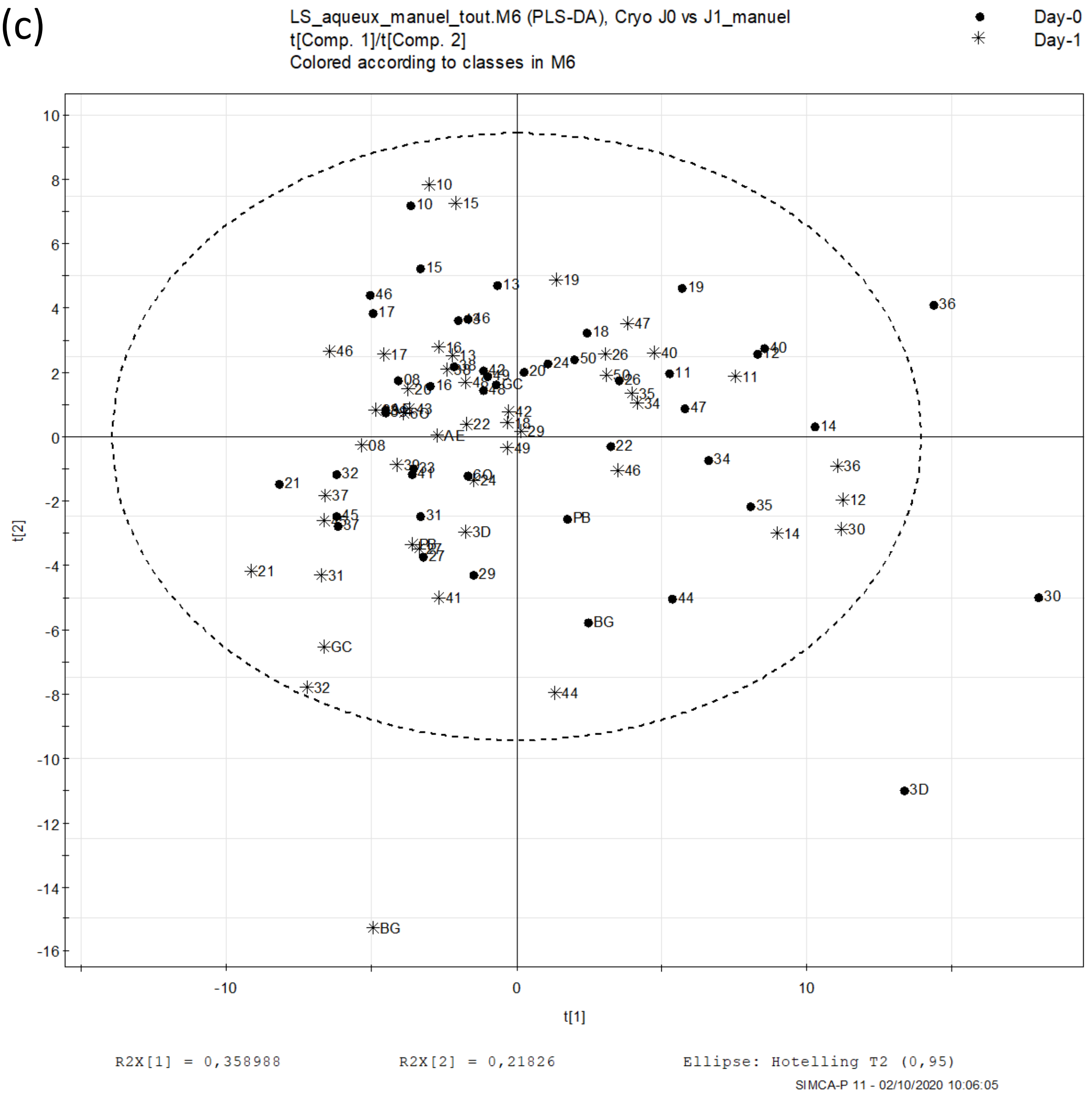

Supplementary data for "1H-NMR-based analysis for exploring knee synovial fluid metabolite changes after local cryotherapy in knee arthritis patients"

(d)

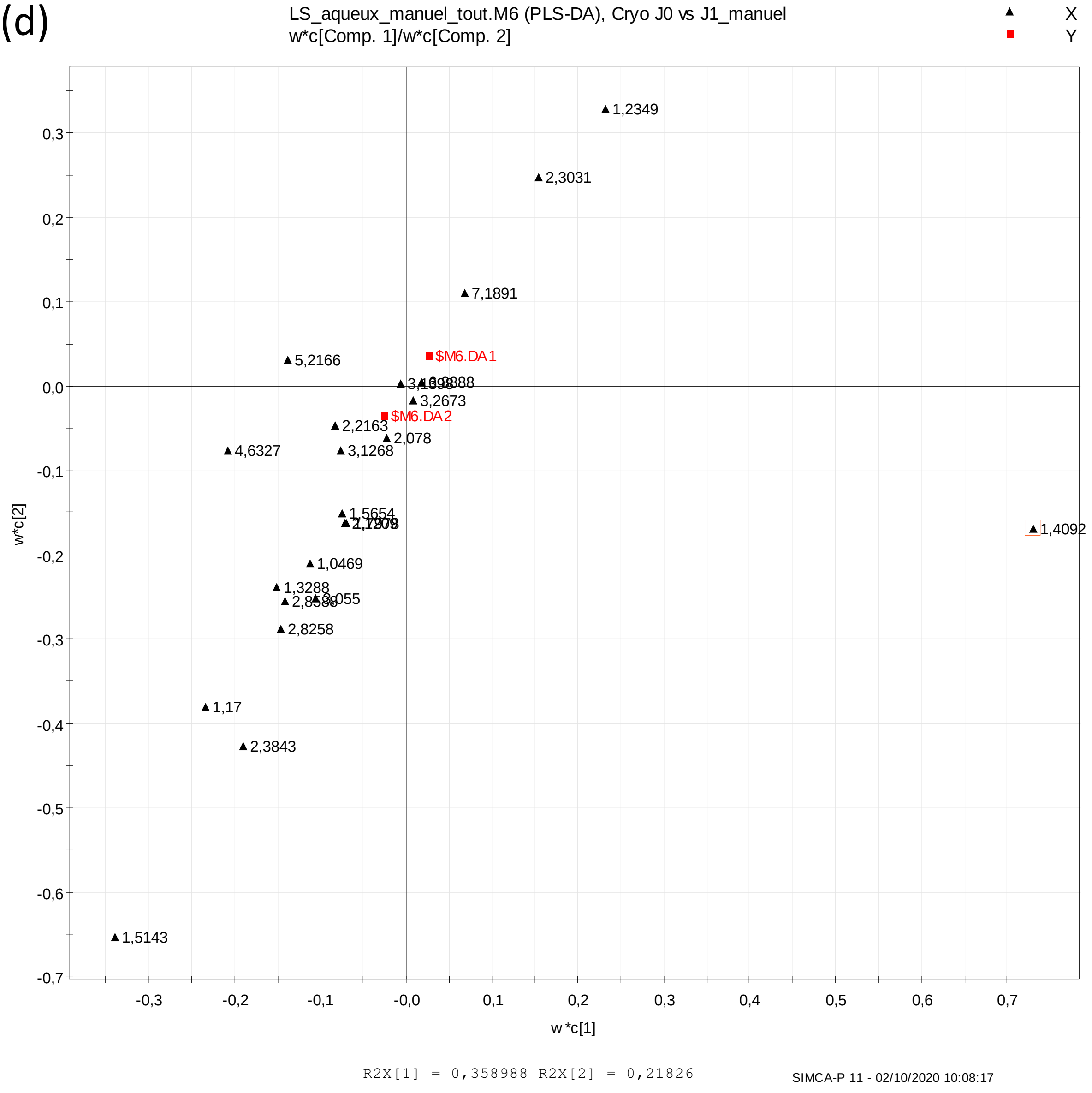

Supplementary data for "1H-NMR-based analysis for exploring knee synovial fluid metabolite changes after local cryotherapy in knee arthritis patients"

SD4 Multivariate analysis results on organic extracts from automatic bucketing

(a) PCA score plot
(b) PCA loading plot
(c) PLS-DA score plot
(d) PLS-DA loading plot

Supplementary data for "1H-NMR-based analysis for exploring knee synovial fluid metabolite changes after local cryotherapy in knee arthritis patients"

(a)

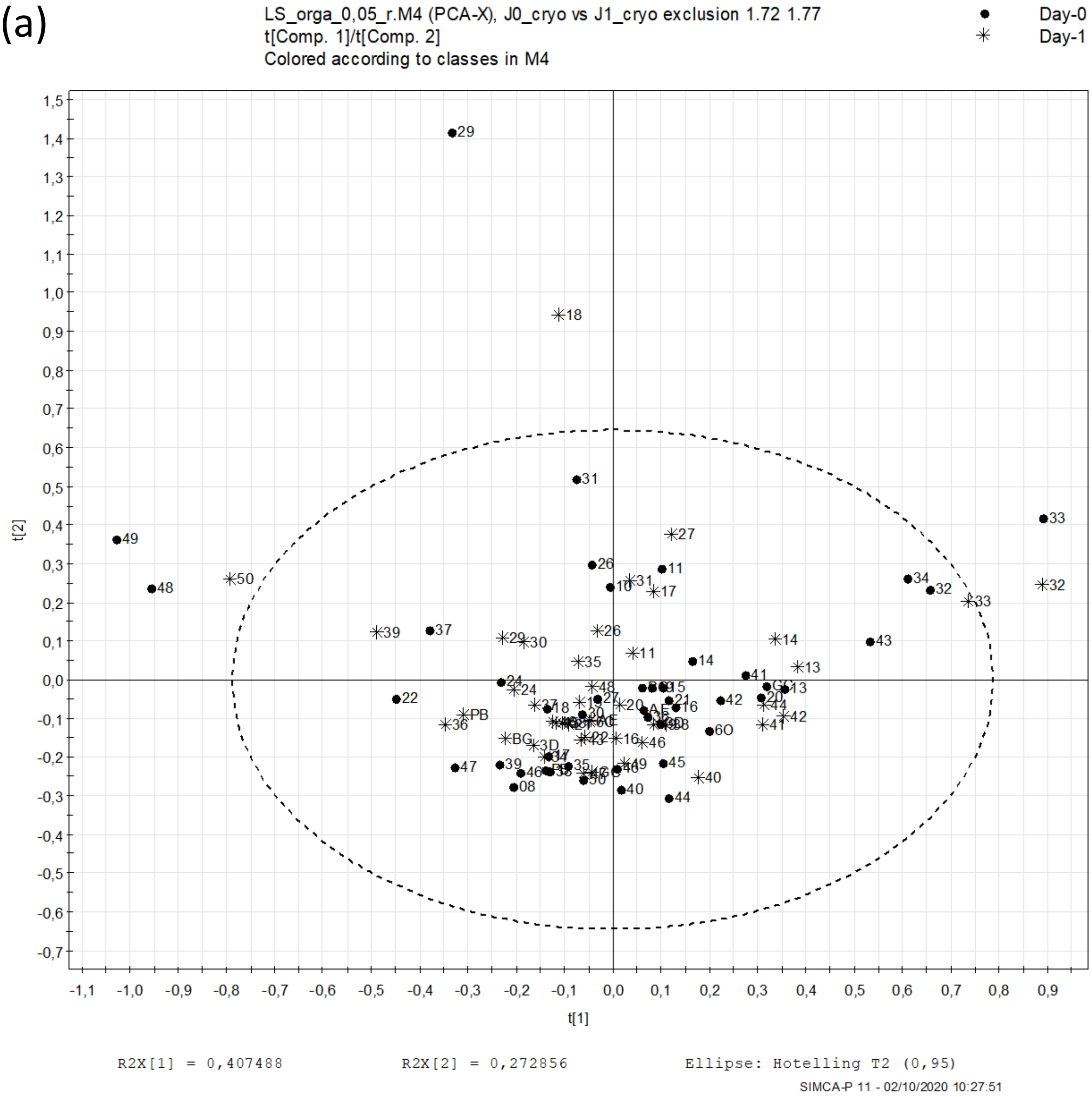

Supplementary data for "1H-NMR-based analysis for exploring knee synovial fluid metabolite changes after local cryotherapy in knee arthritis patients"

(b)

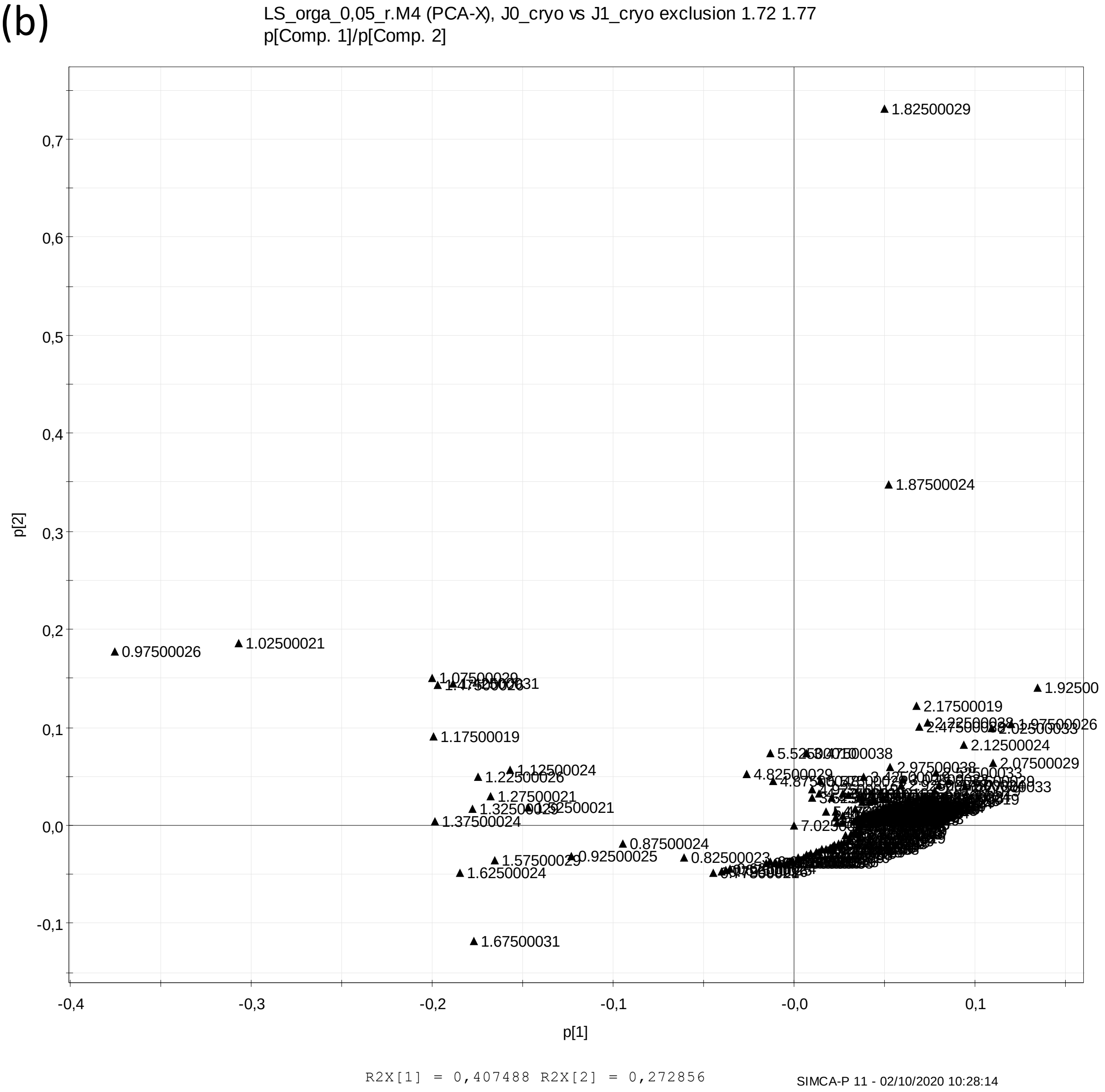

Supplementary data for “1H-NMR-based analysis for exploring knee synovial fluid metabolite changes after local cryotherapy in knee arthritis patients”

(c)

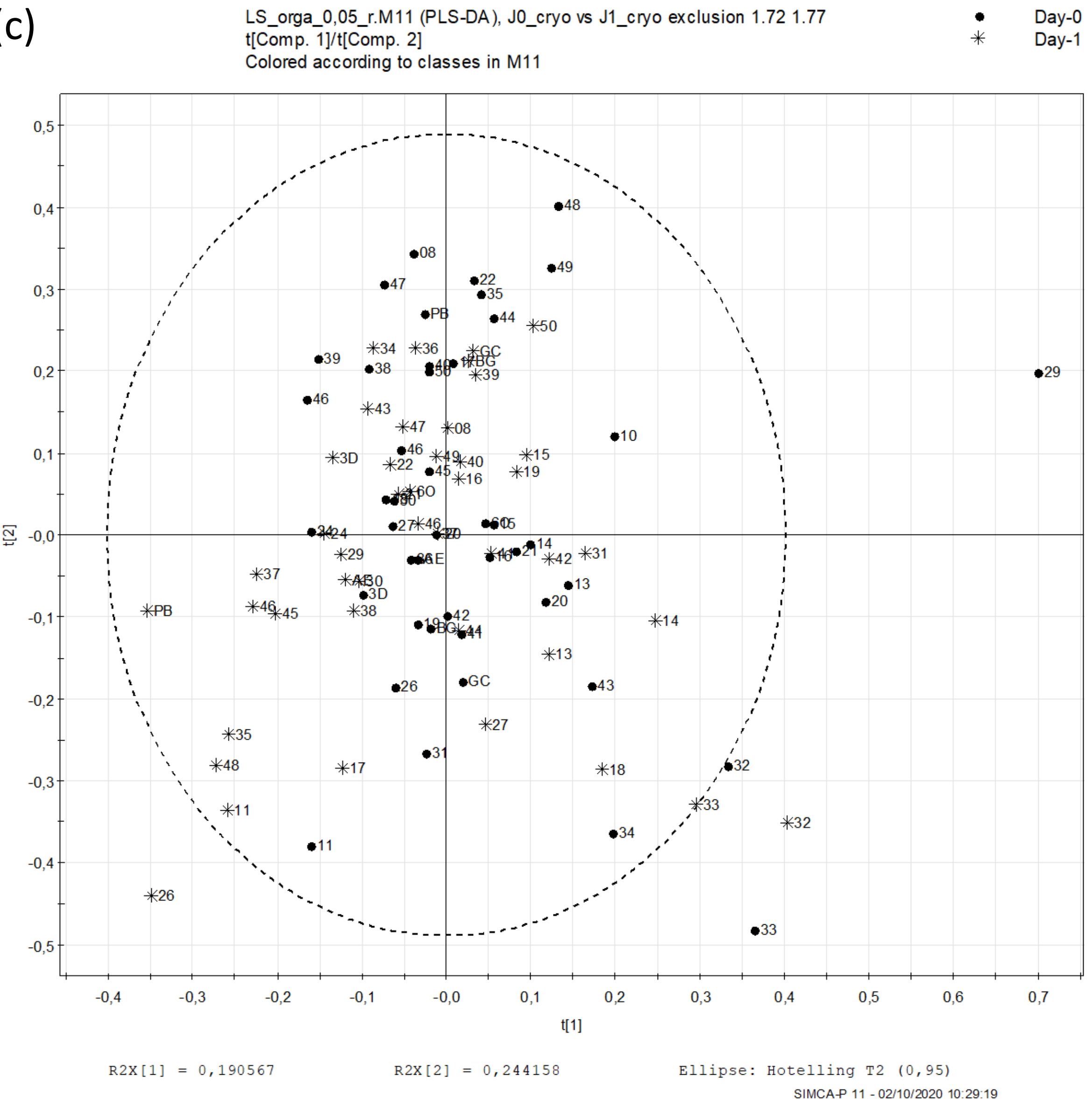

Supplementary data for "1H-NMR-based analysis for exploring knee synovial fluid metabolite changes after local cryotherapy in knee arthritis patients"

(d)

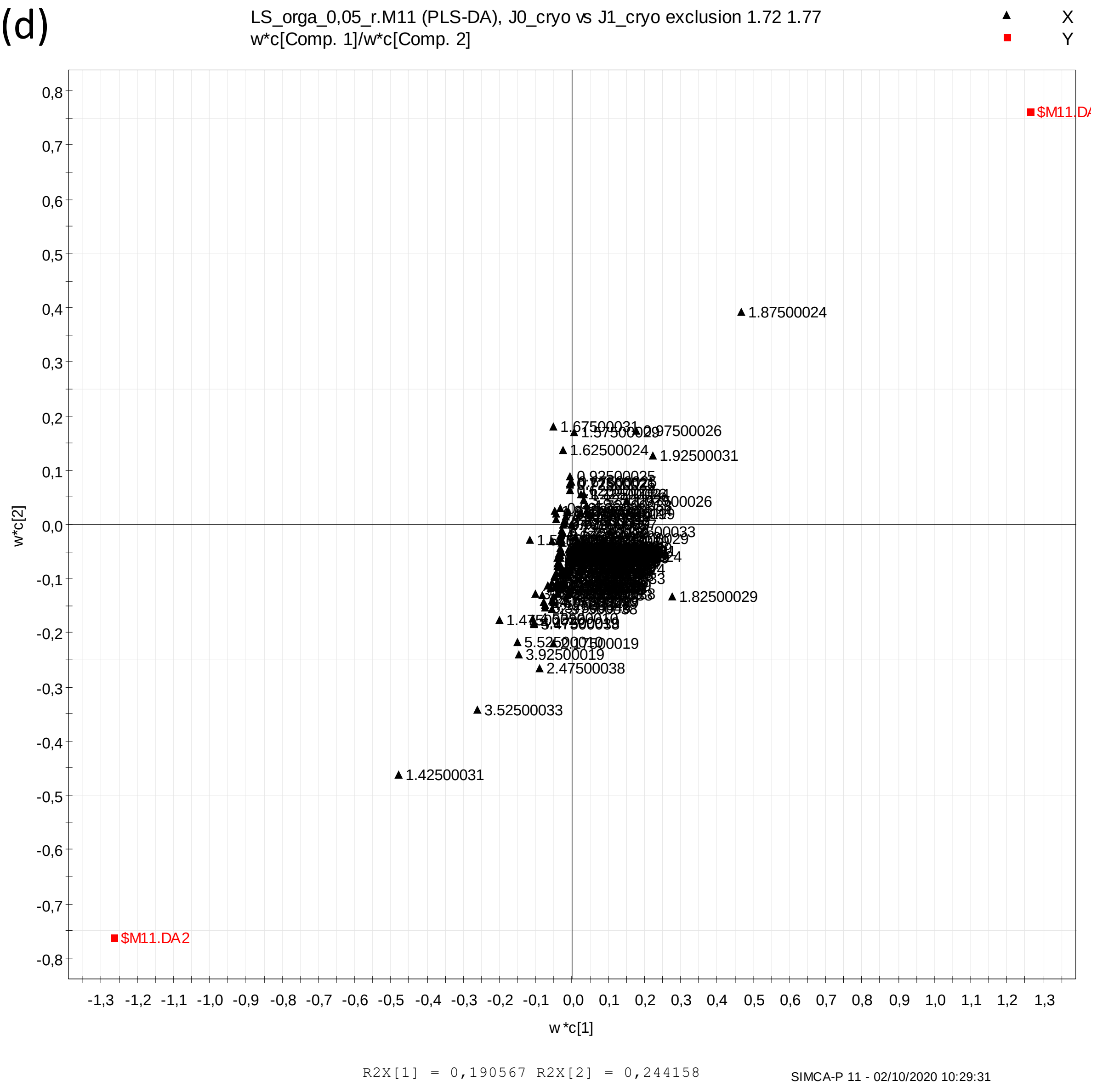

Supplementary data for “1H-NMR-based analysis for exploring knee synovial fluid metabolite changes after local cryotherapy in knee arthritis patients”

SD5 Multivariate analysis results on organic extracts from manual integration

(a) PCA score plot
(b) PCA loading plot
(c) PLS-DA score plot
(d) PLS-DA loading plot

Supplementary data for "1H-NMR-based analysis for exploring knee synovial fluid metabolite changes after local cryotherapy in knee arthritis patients"

(a)

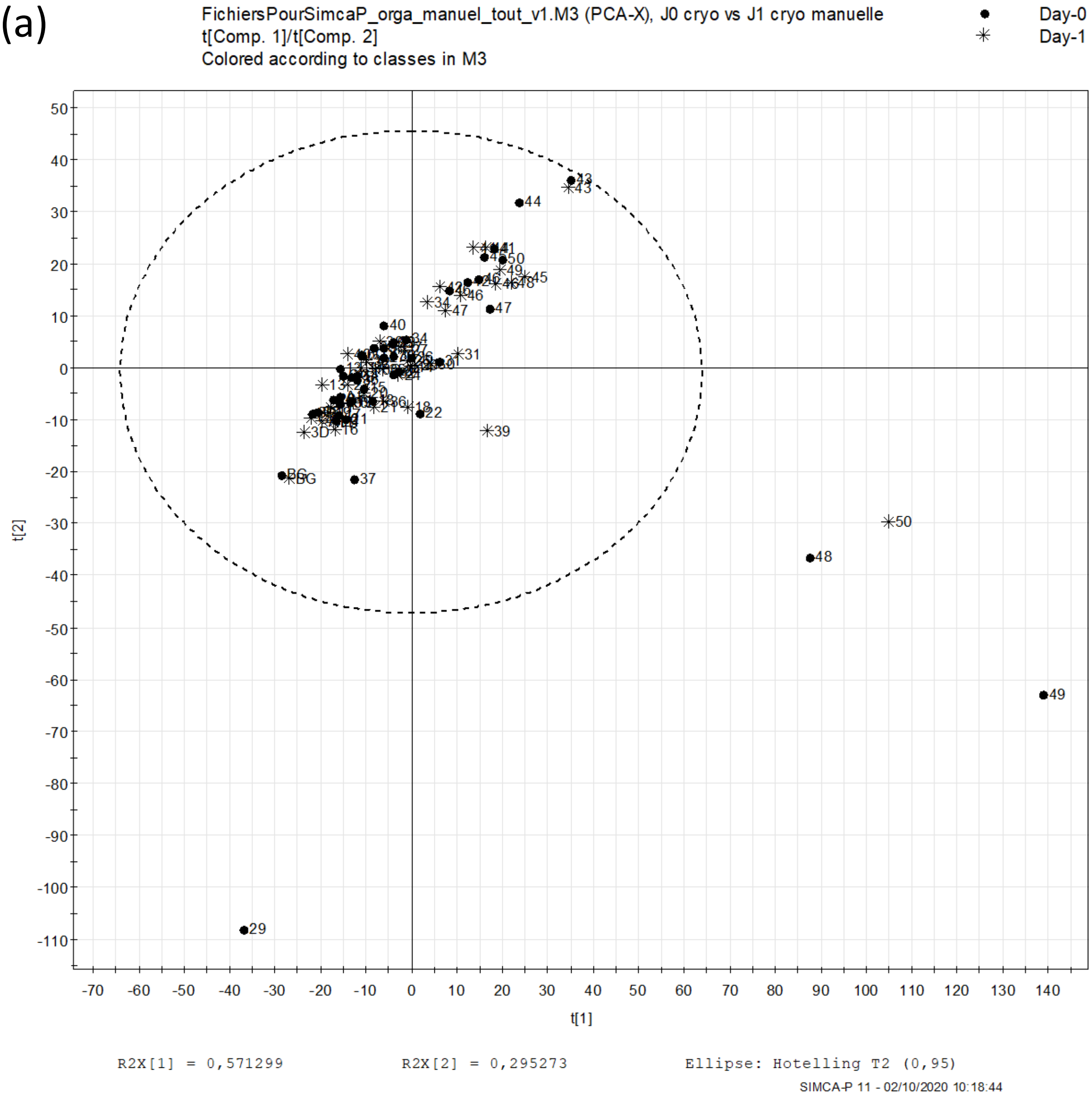

Supplementary data for “1H-NMR-based analysis for exploring knee synovial fluid metabolite changes after local cryotherapy in knee arthritis patients”

(b)

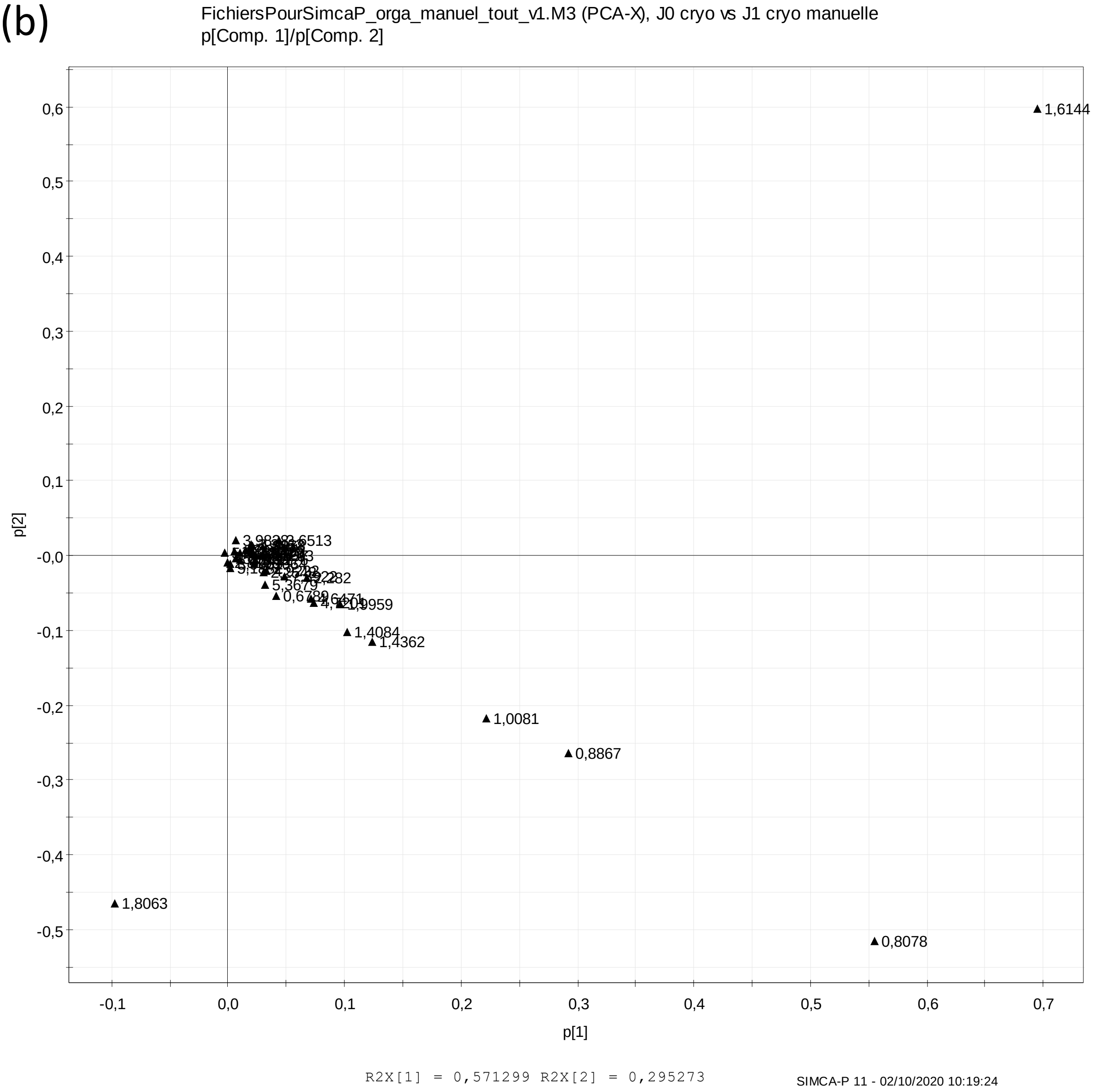

Supplementary data for "1H-NMR-based analysis for exploring knee synovial fluid metabolite changes after local cryotherapy in knee arthritis patients"

(c)

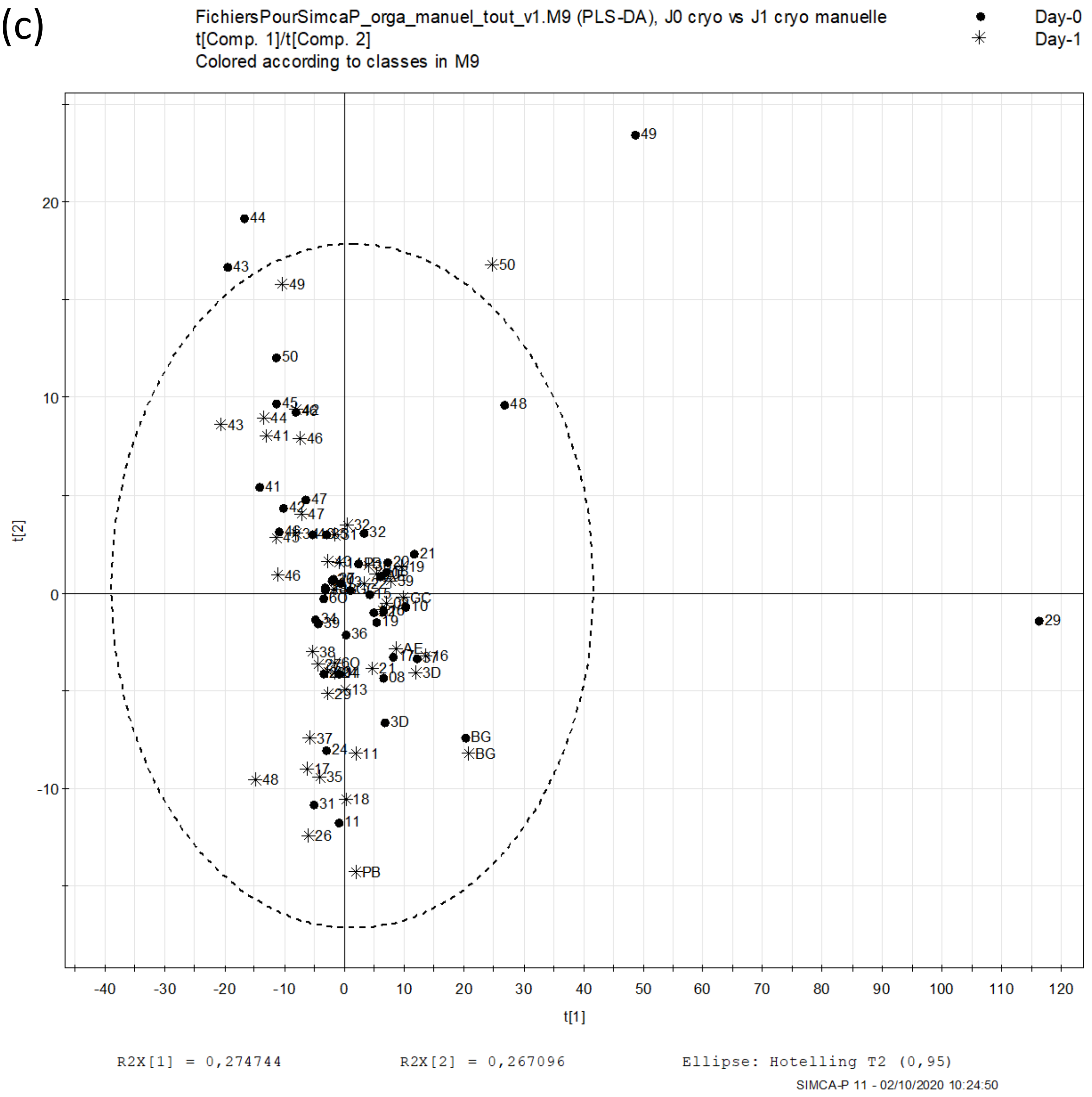

Supplementary data for “1H-NMR-based analysis for exploring knee synovial fluid metabolite changes after local cryotherapy in knee arthritis patients”

(d)

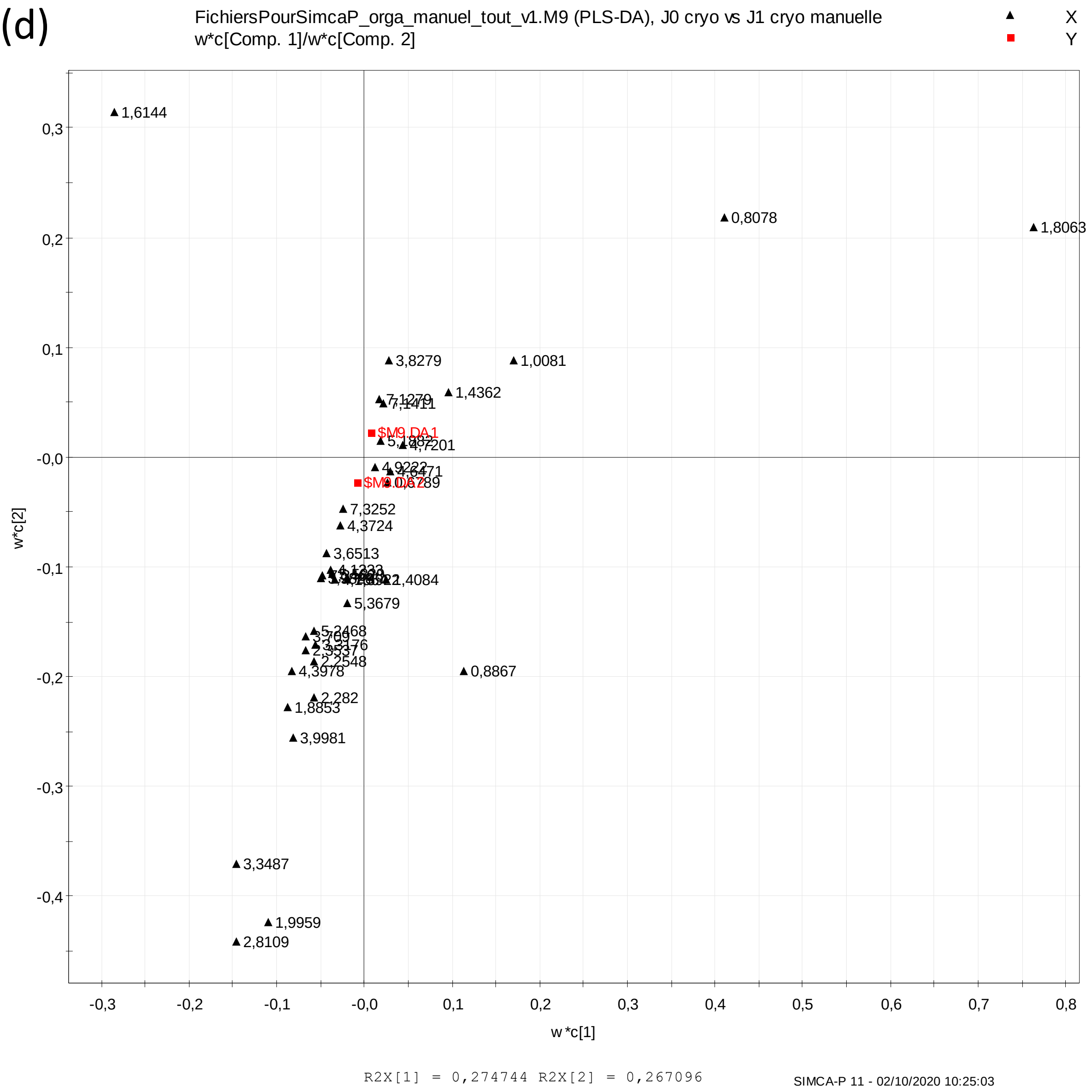

Supplementary data for "1H-NMR-based analysis for exploring knee synovial fluid metabolite changes after local cryotherapy in knee arthritis patients"

SD6 – Comparison of 2 quantification methods: reference (TSP) with sample or reference in a insert

We mixed 3 SF randomly chosen in our bank of samples. The NMR tube preparation was the same as described in experimental methods except the addition of a quantification reference, the TSP. For this last step, we prepared four tube as described below:

TSP with sample

Tube 1a

250 μL of pooled filtrate were mixed with 250 μL of deuterium oxide and 25 μL of deuterium oxide containing TSP.
500 μL were introduced in a 5mm pyrex NMR sample tube.
50 μL of deuterium oxide were added in an insert which was put in the NMR tube.

Tube 1b

250 μL of 3 mM creatine solution diluted as aqueous samples were mixed with 250 μL of deuterium oxide and 25 μL of deuterium oxide containing TSP.
500 μL were introduced in a 5mm pyrex NMR sample tube.
50 μL of deuterium oxide were added in an insert which was put in the NMR tube.

TSP in an insert

Tube 2a

250 μL of pooled filtrate were mixed with 275 μL of deuterium oxide.
500 μL were introduced in a 5mm pyrex NMR sample tube.
25 μL of deuterium oxide and 25 μL of deuterium oxide containing TSP were added in an insert which was put in the NMR tube.

Tube 2b

250 μL of 3 mM creatine solution diluted as aqueous samples were mixed with 275 μL of deuterium oxide
500 μL were introduced in a 5mm pyrex NMR sample tube.
25 μL of deuterium oxide and 25 μL of deuterium oxide containing TSP were added in an insert which was put in the NMR tube.

Quantification of tube 1a was performed relatively to tube 1b and quantification of tube 2a was performed relatively to tube 2b. The results were obtained in accordance with formulae described in experimental section and are presented below. Depending on each metabolite, we observed difference from 0 to 20 %, in a positive or a negative ways; we can think that those differences are mainly due to residual error due to integration and not to binding molecules to TSP.

Supplementary data for "1H-NMR-based analysis for exploring knee synovial fluid metabolite changes after local cryotherapy in knee arthritis patients"

Table SD6. Concentration (mM) of metabolites of one SF sample calculated using two methods : reference mixed with the SF and reference in an insert.

Names	Method		(TSP with sample-TSP in an insert) / TSP with sample
	TSP with sample	TSP in an insert	
Valine	0,68	0,66	3%
Ethanol	0,15	0,14	6%
3-hydroxybutyrate	0,09	0,08	8%
Threonine	0,31	0,34	-12%
Lactate	8,37	8,18	2%
Alanine	0,77	0,69	10%
n-butyrate	0,17	0,16	6%
Acetate	0,03	0,03	17%
NAAG	0,10	0,11	-12%
Methionine	0,09	0,08	13%
Acetone	0,05	0,04	7%
Acetoacetate	0,11	0,10	10%
Pyruvate	0,16	0,15	4%
Citrate	0,06	0,06	2%
Creatinine	0,07	0,07	5%
Dimethylsulfone	0,08	0,08	1%
Ethanolamine	0,08	0,08	-3%
Betaine	0,09	0,09	2%
β-glucose	9,26	10,58	-14%
α-glucose	4,94	5,16	-4%
Tyrosine	0,18	0,18	1%
